# Functional validation of DvABCB1 as a receptor of Cry3 toxins in western corn rootworm, *Diabrotica virgifera virgifera*

**DOI:** 10.1038/s41598-020-72572-9

**Published:** 2020-09-28

**Authors:** Xiping Niu, Adane Kassa, James Hasler, Samantha Griffin, Claudia Perez-Ortega, Lisa Procyk, Jun Zhang, Deirdre M. Kapka-Kitzman, Mark E. Nelson, Albert Lu

**Affiliations:** 1 Corteva Agriscience, 7300 NW 62nd Ave., Johnston, IA 50131 USA; 2 Present Address: Danville, USA; 3 Present Address: Corteva Agriscience, 9330 Zionsville Rd, Indianapolis, IN 46268 USA; 4 Present Address: Reaction Biology Corp, 1 Great Valley Pkwy Ste 2, Malvern, PA 19355 USA

**Keywords:** Entomology, Plant biotechnology, RNAi, Molecular biology

## Abstract

Western corn rootworm (WCR), *Diabrotica virgifera virgifera* (Coleoptera: Chrysomelidae), is a serious insect pest in the major corn growing areas of North America and in parts of Europe. WCR populations with resistance to *Bacillus thuringiensis* (Bt) toxins utilized in commercial transgenic traits have been reported, raising concerns over their continued efficacy in WCR management. Understanding the modes of action of Bt toxins is important for WCR control and resistance management. Although different classes of proteins have been identified as Bt receptors for lepidopteran insects, identification of receptors in WCR has been limited with no reports of functional validation. Our results demonstrate that heterologous expression of DvABCB1 in Sf9 and HEK293 cells conferred sensitivity to the cytotoxic effects of Cry3A toxins. The result was further validated using knockdown of *DvABCB1* by RNAi which rendered WCR larvae insensitive to a Cry3A toxin. However, silencing of *DvABCB2* which is highly homologous to DvABCB1 at the amino acid level, did not reduce the sensitivity of WCR larvae to a Cry3A toxin. Furthermore, our functional studies corroborate different mode-of-actions for other insecticidal proteins including Cry34Ab1/35Ab1, Cry6Aa1, and IPD072Aa against WCR. Finally, reduced expression and alternatively spliced transcripts of *DvABCB1* were identified in a mCry3A-resistant strain of WCR. Our results provide the first clear demonstration of a functional receptor in the molecular mechanism of Cry3A toxicity in WCR and confirmed its role in the mechanism of resistance in a mCry3A resistant strain of WCR.

## Introduction

The western corn rootworm (WCR), *Diabrotica virgifera virgifera* LeConte (Coleoptera: Chrysomelidae), is a key invasive insect pest of maize in the United States and Europe^1–3^. Damage from corn rootworms accounts for over $1 billion in economic impact in North America annually^3,4^. Historically, WCR has been managed by crop rotation and soil insecticides^5–7^ and in 2003 the first *Bacillus thuringiensis* (Bt) toxin-based traits for corn rootworm protection were introduced in the US with the commercialization of Cry3Bb1^8^. Since then three additional Bt toxins (Cry34Ab1/Cry35Ab1 in 2005, mCry3Aa in 2007, eCry3.1Ab in 2012) have been commercialized for WCR management^9–11^. Widespread adoption of Bt crops has helped to decrease the use of broad-spectrum chemical insecticides^12–14^. However, high adoption has also increased the selection pressure for insect pests to develop resistance to Bt traits. Cases of field resistance to Cry3Bb1 and cross-resistance to mCry3A and eCry3.1Ab have been reported in WCR^15,16^. Lab bioassays confirmed cross-resistance among Cry3Bb1, mCry3Aa, and eCry3.1Ab, but not to the binary Bt toxin Cry34/35Ab1^11,17,18^. These cases of field resistance coupled with demonstrated cross-resistance amongst the Cry3-based traits threatens the usefulness of these traits to growers in the US.

A clear understanding of the receptors that are involved in the mode of action of Cry3 toxins can facilitate the development of new technologies for WCR management and provide potential markers for resistance monitoring. Biochemical characterization of a laboratory-selected mCry3A resistant WCR strain revealed reduced binding of mCry3A and the Cry3Aa-like toxin, IP3-H9^19,20^ to brush border membrane vesicles (BBMV) prepared from isolated WCR midguts^21^. A cadherin-like protein was originally identified as a Bt receptor in the lepidopteran *Heliothis virescens*^22^. Subsequently, in Coleoptera, the *Tenebrio molitor* (mealworm) cadherin (TmCad1) was shown to bind Cry3Aa through domain II loop 1 at cadherin repeat 12 (CR12) and to be a functional receptor by RNAi-mediated suppression of its expression^23,24^. *Tribolium castaneum* (red flour beetle) cadherin-like protein (TcCad1) and a sodium solute symporter (TcSSS), which contains a putative binding epitope homologous to cadherin repeats, were demonstrated by RNAi to be functional receptors for Cry3Ba^25^. A high-affinity Cry3 binding site in cadherin repeat 10 (CR10) of DvCad1 protein from WCR (GenBank no. EF531715) enhanced Cry3Bb toxicity to Colorado potato beetle (CPB, *Leptinotarsa decemlineata*) and lesser mealworm (*Alphitobius diaperinus*) neonates^26^, however, loss of DvCad1 expression in WCR using RNAi did not reduce the toxicity of Cry3Aa or Cry34Ab1/Cry35Ab1 indicating that DvCad1 was not a functional receptor for either toxin^27^. Interestingly, ADAM10 metalloprotease which interacts with Cry3Aa on ligand blots has also been demonstrated by RNAi to be a functional receptor of Cry3Aa toxin in CPB^28^. These seemingly disparate results may reflect the complexity of toxin-receptor interactions that occur even in closely related coleopteran species.

Several novel Cry toxin binding proteins have been identified in various insects using in vitro protein–protein interaction techniques (e.g., pull-down, ligand blot, affinity chromatography, etc.) often coupled with tandem liquid chromatography-mass spectrometry (LC–MS/MS) analysis. Examples include actin, vacuolar ATP synthase (V-ATPase) subunits A and B, heat shock cognate protein, polycalin, and prohibitin^25,29–34^. Additional functional validation would provide a better understanding of the role that these interacting proteins might play as bona fide receptors in vivo resulting in toxicity. As an example, a study that evaluated the interactions of Cry3Ba with *T. castaneum* midgut tissue identified five proteins by mass spectrometry that interacted with Cry3Ba in ligand blots, however, only TcCad1 and TcSSS were subsequently validated as functional Cry3Ba receptors using RNAi^25^. Transcript profiling has also been used to find genes potentially responsible for Cry protein toxicity in WCR but this approach requires prior knowledge or assumptions about gene function because a large number of differentially expressed transcripts were found in a transcript profiling experiment^35^. Several protein families including the ATP-binding cassette (ABC) transporters, cadherin, aminopeptidase N (APN), and alkaline phosphatase (ALP) have been identified as receptors of Cry toxins in Lepidoptera where more extensive studies were performed^36,37^. Interestingly, some of these protein classes are differentially expressed in Cry3-resistant WCR or WCR treated with Bt toxins (Cry34/35Ab1, Cry3Bb1, or eCry3.1Ab)^35,38,39^ but have yet to be validated as functional receptors.

Several members of the ABC transporter family have been linked to Cry toxin resistance or shown to be involved in Cry protein toxicity^40^. These proteins constitute a large family of membrane proteins that are found in all organisms and have diverse functions relating to solute transport^41^. Eight ABC transporter subfamilies, designated A through H, have been found in insects^42–44^. In Lepidoptera, the ABCC2 and ABCA2 proteins have been functionally validated as Cry toxin receptors^37,45^. In Coleoptera, ABCB1was identified as a functional receptor for Cry3Aa in leaf beetle (*Chrysomela tremula*) using genetic linkage to resistance coupled with heterologous expression of the wild type gene to confer Cry3Aa sensitivity to Sf9 cells^46^. Based on these reports we identified a putative WCR orthologue, ABCB1, and the highly homologous sequence, ABCB2, to characterize and understand whether either or both serve similar roles in mediating Cry3 toxicity in this important pest of maize. We validated DvABCB1 as a functional receptor to Cry3A in WCR using cell-based assays and RNAi suppression in vivo, while suppression of *DvABCB2* did not affect Cry3A toxicity. Moreover, our study demonstrates that DvABCB1 is not a functional receptor for IPD072Aa, a new WCR-active toxin, corroborating its lack of cross-resistance in a mCry3A resistant strain of WCR^47^. Finally, reduced expression and alternative splicing of *DvABCB1* transcripts were identified in mCry3A resistant WCR explaining the reduced binding of mCry3A that was previously reported^21^.

## Results

### Identification and predicted structures of DvABCB1 and DvABCB2

A BLAST (tblastx) search of the CtABCB1 sequence against a WCR transcriptome identified a sequence with 69% overall identity at the amino acid level^46,48^. The top hit, Dvv-isotig11620^46^, encodes a 1256 amino acid protein, designated DvABCB1, with a predicted size of 138 kDa. The second best hit, Dvv-isotig07787^46^, encodes a protein consisting of 1257 amino acids that was only 63% identical to CtABCB1 and was therefore designated DvABCB2. Dvv-isotig07787 or DvABCB2 is identical to the sequence found within a 20 cM genomic region that included multiple candidate genes linked to Cry3Bb1 resistance^49^. The identity of DvABCB1 to DvABCB2 is 67% which is lower than its identity to CtABCB1. This may indicate orthologous origination of these genes within Coleoptera.

The suite of InterProScan protein bioinformatics tools (e.g., PROSITE, PRINTS, Pfam, ProDom, SMART, TIGRFAMs, PIR) indicate that DvABCB1 and DvABCB2 are canonical full ABC transporter proteins and highly homologous to the CtABCB1 protein. These transporters are arranged structurally into two half transporter regions, each half having a transmembrane domain (TMD) consisting of 6 transmembrane alpha helices (TMs), three extracellular loops (ECLs), and a cytoplasmic nucleotide binding domain (NBD; Supplementary Fig. 1a). The cytoplasmic loop between the two halves contains the most variable region in addition to its NBD. Among ABCB-type transporters, ECLs 1, 3, 4, and 6 show higher divergence than the smaller ECLs 2 and 5 (Supplementary Fig. 1b). The higher divergence in ECLs 1, 3, 4, and 6 is consistent with their potential as differential binding recognition sites that were reported for other ABC subfamilies utilized by some lepidopteran-active Cry toxins^37^.

### Heterologous expression of DvABCB1 in Sf9 and HEK293 cells confers cell toxicity to Cry3A toxins

**Figure 1:**
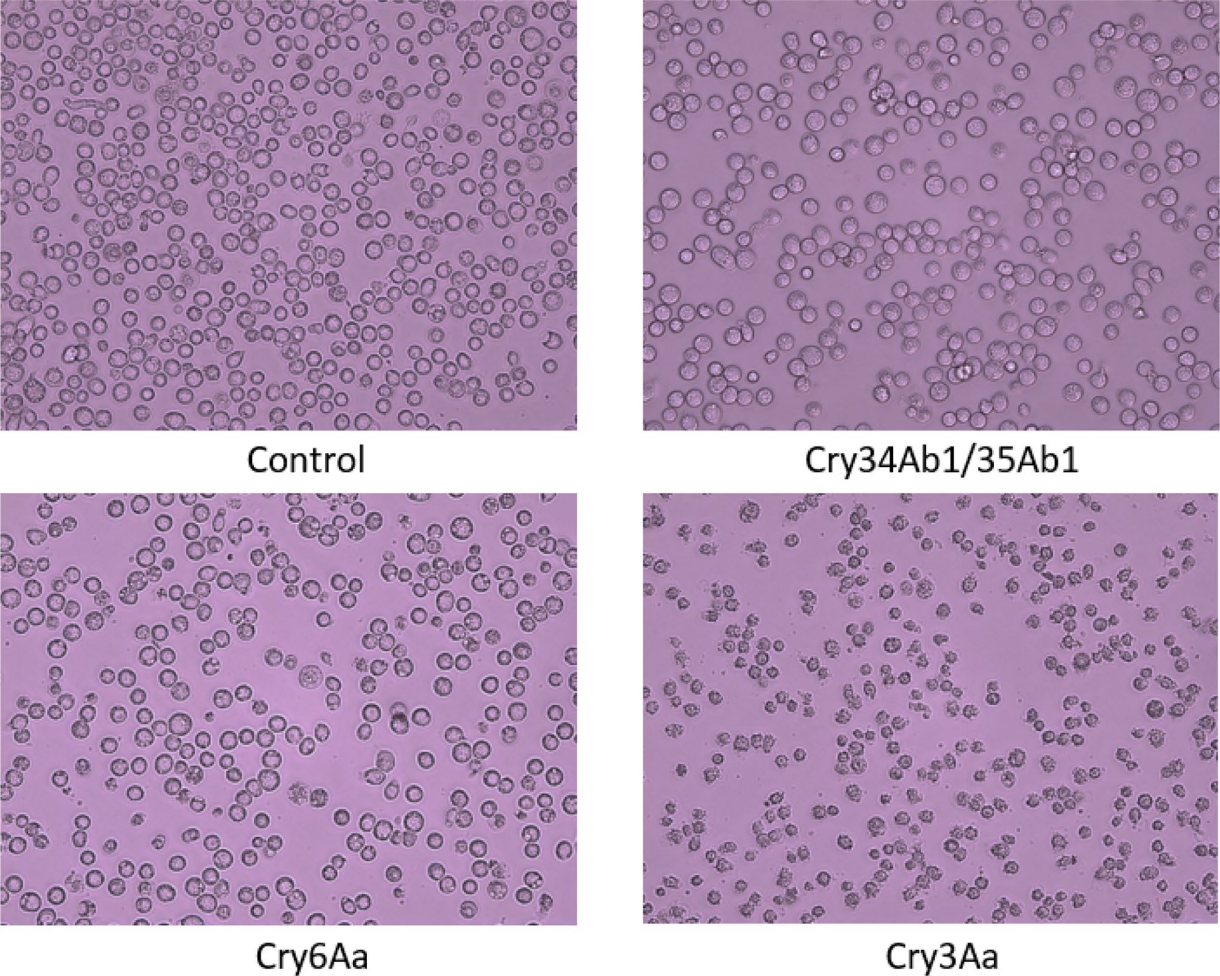
Functional evaluation of DvABCB1 by heterologous expression in Sf9 Cells. Sf9 cells were infected with recombinant baculovirus stock to express DvABCB1. One day after infection, the cells were treated with different protein toxins and medium control as indicated below each panel at 1 µg/mL. After overnight incubation, the cells were imaged.

Cell-based assays have been used to evaluate the functional role of insect toxin receptors^50^. We infected Sf9 cells in culture at a high titer with recombinant baculovirus expressing DvABCB1 to investigate and characterize the effect of subsequent addition of Cry toxins on Sf9 cell toxicity/viability. The toxins tested included Cry3Aa, Cry34Ab1/35Ab1, and Cry6Aa1, all of which were prepared and activated as previously described^51,52^. Cry6Aa1 is another Cry protein that is also highly active against corn rootworms^52^. The cells were evaluated for morphological changes by phase contrast light microscopy after overnight incubation with activated toxins. Nearly complete cell death was observed for cells exposed to Cry3Aa treatment but no effect was observed in cells exposed to either Cry34Ab1/Cry35Ab1 or Cry6Aa1 treatments. Cell morphology of DvABCB1 expressing cells was similar to control cells that were exposed to media only (Fig. 1). These results indicate that heterologous expression of DvABCB1 in Sf9 cells confers cell toxicity selectively to Cry3Aa, but not to Cry34Ab1/35Ab1 or Cry6Aa1.

**Figure 2:**
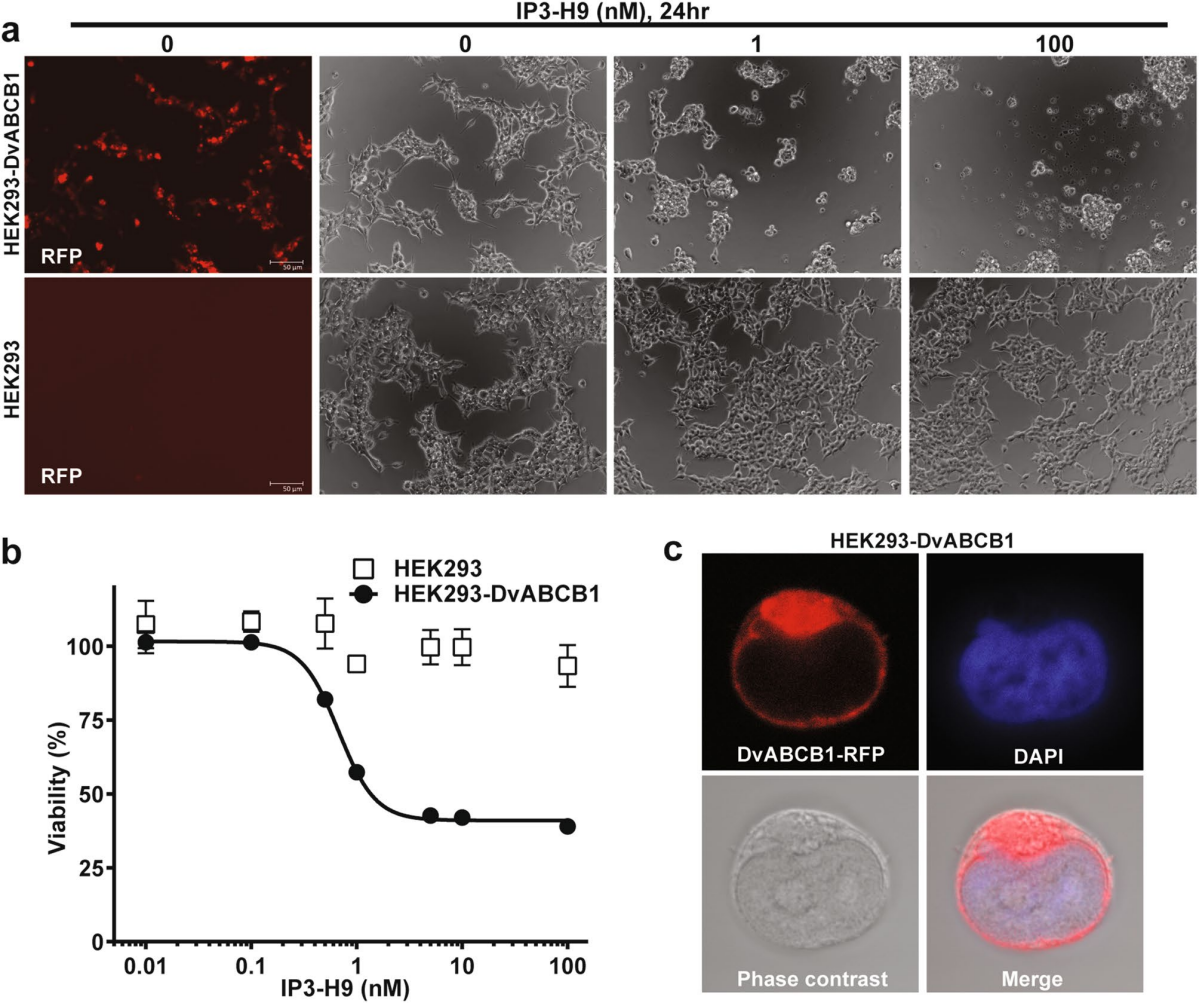
Functional evaluation of DvABCB1 by heterologous expression in HEK293 cells and quantitation of cell toxicity to IP3-H9. (**a**) Dose-dependent morphology change of HEK293 cells expressing Dv-ABCB1 after 24 h of IP3-H9 treatment. HEK293 cells were transfected with DvABCB1 vector and selected with 1 mg/mL of Geneticin for 4 weeks before treated with 0–100 nM of IP3-H9. Expression of DvABCB1-RFP fusion protein was shown as an indicator of DvABCB1 positive cells (left panel, 0 nM IP3-H9 as a representative). (**b**) Cytotoxicity of IP3-H9 to the HEK293 cells expressing DvABCB1. Cells were seeded in 96-well plates overnight before cultured with 0, 0.01, 0.1, 0.5, 1, 5, 10 and 100 nM of IP3-H9 for 24 h. The ATP level of metabolically active cells were quantified by the CellTiter-Glo Viability assay. The luminescence of 10 µM oligomycin challenged cells was subtracted from that of the toxin treated cells as the background absorbance. The cell viability was normalized by using 0 nM IP3-H9 treated cells as 100%. n = 3. (**c**) DvABCB1-RFP (red) was expressed on the cellular surface of HEK293 cells. Cell nuclei were stained with DAPI (blue).

To characterize DvABCB1 functionality more quantitatively, DvABCB1 was expressed in HEK293 cells to establish a stable line. The expression vector for the DvABCB1 cDNA included a fluorescent tag to aid in cloning and characterization of expression. When examined under a confocal microscope, fluorescence from DvABCB1-RFP was observed on the cell surface of HEK293 cells (Fig. 2c). Treatments of DvABCB1-RFP expressing cells with increasing doses of Cry3Aa-like toxin, IP3-H9, led to dose-dependent cell death, while non-transfected cells did not respond to IP3-H9 exposure (Fig. 2a). IP3-H9 is a modified version of Cry3A that has improved solubility compared to Cry3A and is highly cross-resistant to Cry3A when tested in artificial diet bioassays against a Cry3A-resistant strain of WCR^19–21^. Quantifying the cell response revealed about 70% cell mortality under 100 nM toxin treatment with a half maximal response at a concentration of approximately 0.68 nM (EC50 value) (Fig. 2b). The remaining 30% of metabolically active cells may not express sufficient levels of DvABCB1 receptor to result in cell death with toxin exposure as shown in Fig. 2a (the top left panel). These results are similar to the observations reported previously in assays of Sf9 cells expressing certain ABC transporter proteins^46,53^ and demonstrate that DvABCB1 from WCR can also serve as a Cry3A receptor when expressed in HEK293 cells.

### Knockdown of *DvABCB1* by RNAi Renders WCR larvae insensitive to the Cry3A-like toxin, IP3-H9

**Figure 3:**
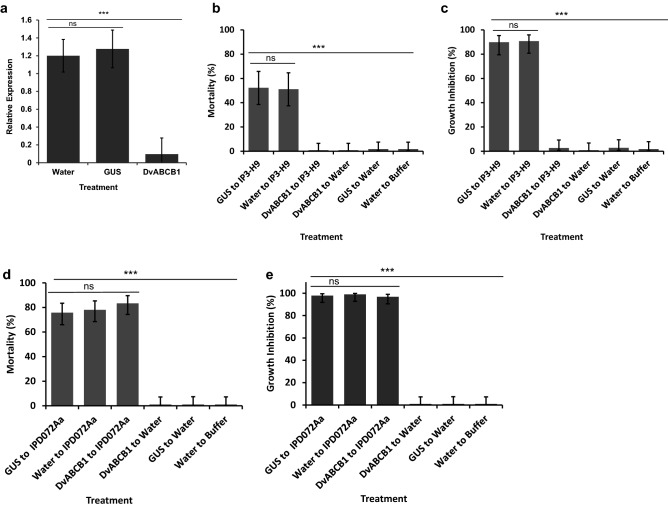
Effect of RNAi suppression of *DvABCB1* on *D. virgifera virgifera* larvae exposed to WCR toxins. WCR neonates were treated with test dsRNA samples [100 µg/mL in diet or water (control)] for 2 days and transferred to the same treatments that were freshly prepare for two more days. Treated larvae were then exposed to protein toxins at the doses equivalent to LC80-85. The effects of treatments on WCR larvae were scored 14 days after initial treatments. Least square means pairwise comparison *p* values: > 0.05 ns (not significant), < 0.0001***. (**a**) Gene suppression analysis of WCR larvae 4 days after dsRNA treatment [mean ± SE; n = 9–12 per treatment (5–6 insects per treatment and n = 2 PCR sub samples per insect)]. Relative expression of *DvABCB1* by qRT-PCR assay is shown for each treatment using *DvRPS10* as a reference and normalized to *DvABCB1* expression in water control. F (2, 30) = 12.37, *p* value < 0.001. Least square means pairwise comparison *p* values: > 0.05 ns (not significant), < 0.001***. (**b**,**c**) Exposure to IP3-H9 after dsRNA treatment: Mortality (F (5, 66) = 15.88, *p* value < 0.001, and growth inhibition (F (5, 66) = 20.89, *p* value < 0.001). (**d**,**e**) Exposure to IPD072Aa after dsRNA treatment: Mortality (F (5, 66) = 19.54, *p* value < 0.001, and growth inhibition (F (5, 66) = 27.34, *p* value < 0.001).

The functional role of DvABCB1 in Cry3A toxicity to WCR larvae was further validated using RNAi to suppress its expression and to demonstrate subsequent insensitivity of treated WCR to Cry3A exposure. A 155 bp double-stranded RNA (dsRNA) was designed to target a region near the 5′ end of *DvABCB1.* The assay was performed with 24 h old WCR larvae using a two-stage artificial diet bioassay. In stage 1 (4 days in length) the larvae were exposed to a high dose^54^ of dsRNA (100 µg/mL of diet) to effectively silence *DvABCB1.* During stage 2 these larvae were transferred to fresh diet and exposed to WCR-active toxins for 10 days. In stage 1 the controls included dsRNA corresponding to *GUS* (β-glucuronidase) and water alone. Also, the larvae were exposed to dsRNA for 2 days and then transferred to fresh dsRNA-containing diet for another 2 days of exposure to counter dsRNA degradation that occur as the larvae feed on the diet^55^. qRT-PCR was used to assess the efficiency of silencing achieved by the 4-day exposure to *DvABCB1* dsRNA during stage 1. The qRT-PCR results showed that more than 90% suppression of the *DvABCB1* transcript was achieved indicating that it was suppressed very effectively as compared to the transcript levels observed for both water and *GUS* dsRNA treatments (Fig. 3a) validating the stage 1 exposure conditions. The stage 2 treatments included individual exposure to IP3-H9, IPD072Aa, buffer, or water controls. IPD072Aa is a new WCR-active toxin that kills mCry3Aa or Cry34Ab1/Cry35Ab1 resistant WCR larvae as effectively as non-resistant strains^47^. Preliminary bioassays were conducted to characterize the sensitivity of untreated larvae to IP3-H9 and IPD072Aa during stage 2 exposure to determine an appropriate dose to use for toxicity testing (Supplementary Table 1). Stage 2 exposure to IP3-H9 (200 µg/mL) caused about 51–52% (95% CL 37–66%) mortality and 90% (95% Cl 79–96%) growth inhibition effect (see “Materials and methods” section) to larvae that were pre-exposed to *GUS* dsRNA or water controls in stage 1 (Fig. 3b,c). Similarly, stage 2 exposure to IPD072Aa caused high mortality (76–86%; 95% CL 66–85.4%) and growth inhibition (98–99%; 95% CL 82–100%) to larvae that were pre-exposed to *GUS* dsRNA or water in stage 1 (Fig. 3d,e). Mortality and growth inhibition for stage 2 negative controls were under 5% suggesting prior exposure to *DvABCB1* or *GUS* dsRNA did not impact larval growth and development (Fig. 3b,c).

Significant differences, however, were observed in responses between larvae that were pre-treated with *DvABCB1* dsRNA during stage 1 when exposed to IP3-H9 and IPD072Aa in stage 2. No significant mortality or growth inhibition was observed for larvae exposed to IP3-H9 (200 μg/mL) similar to that observed for the water or buffer controls (Fig. 3b,c). In contrast, larvae responded to IPD072Aa treatment (200 μg/mL) in a similar way as those that were exposed to either *GUS* dsRNA or water during stage 1 (Fig. 3d,e). These results demonstrate that *DvABCB1* is a functional receptor for Cry3A toxins such as IP3-H9, but not for IPD072Aa. To our knowledge this is the first report that clearly demonstrates and validates a functional role in WCR for a receptor for Bt toxins that have been deployed commercially for control of damage caused by this pest.

**Figure 4:**
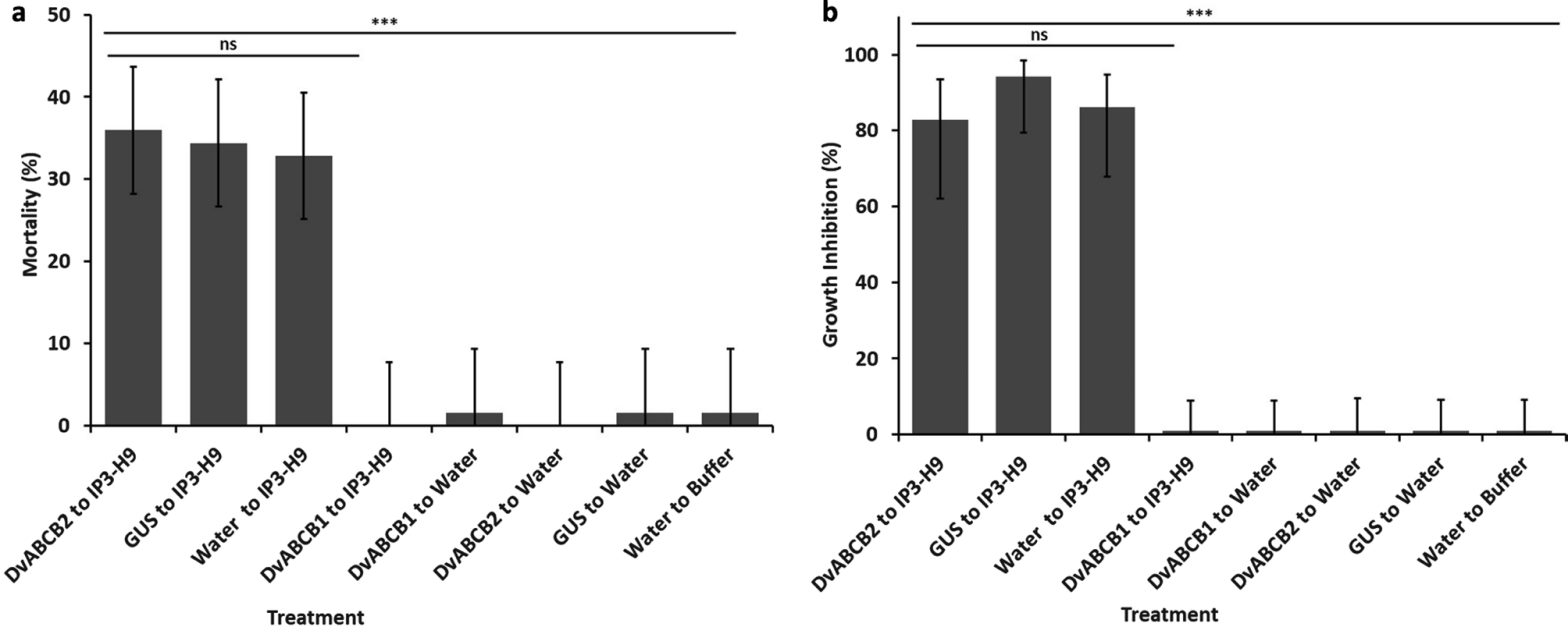
Effect of RNAi suppression of *DvABCB2* on *D. virgifera virgifera* larvae exposed to IP3-H9. WCR neonates were treated with test dsRNA samples [100 µg/mL in diet or water (control)] for 2 days and transferred to the same treatments that were freshly prepare for two more days. Treated larvae were then exposed to IP3-H9 at 200 µg/mL in diet. The effects of treatments on WCR larvae were scored 14 days after initial treatments. Least square means pairwise comparison *p* values: > 0.05 ns (not significant), < 0.0001***. (**a**) Mortality (F _(7, 56)_ = 20.15, *p* value < 0.001, percent mortality data was analyzed using general linear mixed model with Gaussian distribution with identity function and the Laplace likelihood approximation method. (**b**) Growth inhibition (F _(7, 56)_ = 9.90, *p* value < 0.001). Growth inhibition (dead plus severely stunted larvae/total exposed) data were subjected to statistical analysis using general linear mixed model with a binomial distribution, a logit link function, and the Laplace likelihood approximation method.

A previous study implicated a different ABCB-type transporter which we have designated DvABCB2 in resistance to Cry3 in field populations of WCR^49^. To investigate a role for DvABCB2 in Cry3A toxicity, a second RNAi study was performed where stage 2 exposure to IP3-H9 caused 36% (95% CL 28–44%) mortality and 83% (95% Cl 62–93.5%) growth inhibition to larvae that were pre-exposed to *DvABCB2* dsRNA in stage 1 (Fig. 4a,b). Furthermore, these levels of mortality and growth inhibition were statistically similar with stage 2 IP3-H9 exposure of *GUS* dsRNA or water pre-exposed treatments in stage 1 (Fig. 4a,b). Generally, the observed mortality due to stage 2 IP3-H9 exposure of *GUS* dsRNA or water treatments in stage 1 was lower than the first study. Nonetheless, our results show that in contrast to *DvABCB1*, silencing of *DvABCB2* did not reduce the sensitivity of WCR larvae to IP3-H9. Moreover, prior exposure to *DvABCB2* dsRNA did not impact larval growth and development, as was the case for larvae exposed to *DvABCB1* dsRNA. *DvABCB2* dsRNA was designed to target toward the 5′end of the gene specifically where *DvABCB1* and *DvABCB2* are highly divergent (no 21-mer with more than 81% identity at the nucleotide level). This level of identity is below the threshold needed for efficient RNAi suppression of a non-target gene^56^. qRT-PCR assays indicate *DvABCB2* transcript was knocked down specifically by *DvABCB2* dsRNA, but not by *DvABCB1* dsRNA or vice versa (Supplementary Fig. 2a and b).

### *DvABCB1* expression is altered in mCry3A-resistant WCR

Since silencing of *DvABCB1* by RNAi was sufficient for eliminating the response of WCR larvae to IP3-H9, we decided to evaluate if alterations in *DvABCB1* could be responsible for the high resistance and reduced binding of IP3-H9 in a mCry3-resistant strain of WCR (Zhao et al.^21^). RT-PCR amplicon sequencing confirmed the presence of full length *DvABCB1* transcript (3771 bp) in the susceptible strain of WCR (with 11 SNPs; excluding nt position 21, SNPs as reported in cell-based assay cloning section). For the mCry3A-resistant strain, *DvABCB1* amplicon sequences were identified and designated *DvABCB1_3AR1* (3045 bp) and *DvABCB1_3AR2* (1341 bp), as well as a full-length transcript (with 14 silent SNPs and one nonsynonymous SNP at nt position 1501:T to G, changing F to V. Supplementary Fig. 3). Alignments of the nucleotide and amino acid sequences from the susceptible and resistant strains show that *DvABCB1_3AR1* and *DvABCB1_3AR2* contain large deletions (726 bp and 2430 bp, respectively) with no frame shifts or stop codons (Supplementary Fig. 4). Aligning the *3AR1* and *3AR2* sequences using a WCR genomic model sequence (NCBI Dvir_v2.0; Accession No. NW_021043569) and the identified *DvABCB1* nucleotide sequence (TBLASTN of DvABCB1 in NCBI), revealed that the start of the deleted sequences occurred after annotated exon 7 of LOC114344372, at the exon–intron junction. The *DvABCB1_3AR1* sequence resumes at the beginning of annotated exon 11 of LOC114344372, which is the last annotated exon in the model (data not shown). NW_021043569 contains only the partial sequence of *DvABCB1* (the later part of the gene sequence is missing) and does not permit identification of the exon where the *DvABCB1_3AR2* sequence resumes. Regardless, the location of the deletions at exon–intron junctions provides a strong indication that the resistant sequences are products of alternative splicing. More importantly to the mechanism of Cry3 resistance, the extent of the deletions would be predicted to eliminate key regions of the protein which would have significant impact on the folding and membrane topology (both sequences lack predicted TM6 and NBD1) such that the binding of Cry3 proteins would be adversely affected.

Finally, analysis by qRT-PCR indicates that expression of *DvABCB1* transcripts in the mCry3A resistant strain was only 23% of that in susceptible insects (Supplementary Fig. 5). Reduced DvABCB1 transcript levels in Cry3A resistant insects is in agreement with suppression of *DvABCB1* by RNAi in susceptible insects to render WCR larvae insensitive to Cry3A-like toxin.

## Discussion

WCR is one of the most economically impactful insect pests of maize production in North America and Europe and has proven to be highly adaptive to pest management practices^1–3^. Bt traits that target WCR have been an effective tool available to growers in North America for over 15 years, but instances of field resistance to the individual trait proteins have been reported^57^. A better understanding of the mode of action of insecticidal traits will provide knowledge of how resistance may develop and may also provide useful molecular tools to track resistance alleles in field populations to help inform resistance management strategies. Field and lab studies have revealed cross-resistance among all commercialized varieties of Cry3 toxins present in today’s rootworm traits^11,15^ strongly suggesting a shared step in their modes of action. Mutations or changes in expression of a common receptor may explain the cross resistance but the identity of functional Cry3 receptor(s) in WCR has not been elucidated previously. Through gain- and loss-of-function studies, the results presented here demonstrate that DvABCB1 is a receptor for Cry3A toxins.

Expression of CtABCB1 in stable clones of Sf9 cells has been previously used as an indirect demonstration of its functional role in Cry3Aa toxicity in leaf beetle^46^. Similarly, we have shown that Cry3A cytotoxicity is promoted in Sf9 cells infected with recombinant baculovirus expressing DvABCB1. Direct support for the in vivo role of DvABCB1 in WCR Cry3A toxicity was established by using RNAi to reduce expression of *DvABCB1* in the midgut of WCR larvae. Our results demonstrated that suppression of *DvABCB1* expression in WCR larvae eliminated WCR toxicity to Cry3A toxin, IP3-H9, but had no effect on the activity of a new rootworm active, IPD072Aa, isolated from *Pseudomonas chlororaphis*. In Lepidoptera, ABCC2 has been shown to be central to the mode of action of certain Cry1A toxins, but cadherin also contributes to Cry1A toxicity when expressed in cell lines^53^. This finding is consistent with the role that cadherin plays in many examples of Cry1A resistance^58^. Similarly, while DvABCB1 may play an important role in Cry3A toxicity in WCR other proteins such as cadherin and ADAM10 metalloprotease have been implicated for their role as Cry3 receptors in different coleopteran insects^23–25^. In the case of cadherin, this putative Cry3A receptor in WCR was not validated by in vivo testing^27^. Our finding that suppression of *DvABCB1* in WCR by RNAi has such a profound effect on Cry3A susceptibility suggests that in WCR it is a functional Cry3A receptor. Whether other putative receptors such as ADAM10 metalloprotease or a sodium solute transporter, which have been implicated as Cry3 interacting proteins in other *Coleoptera*^24–26,28^, are functional receptors remains to be determined.

Our finding that DvABCB1 rather than DvABCB2 is a major receptor of Cry3Aa is based on how the *DvABCB1* or *DvABCB2* dsRNA was specifically designed to target 5′ gene regions with low homology between *DvABCB1* and *DvABCB2*. This strategy significantly reduced possible off-target effects between them based on bioinformatic analysis indicating that there are no 21-mers with greater than 81% identity (at least 4 bp differences) that should be generated as a result of *DvABCB1* or *DvABCB2* dsRNA treatment^56^. Similar to the situation in leaf beetles where two closely related *ABCB* genes have been identified, only a mutant form of CtABCB1 was linked to Cry3Aa resistance^43,46^. Furthermore, while ABCC2 proteins have been identified as Cry1 toxin receptors in Lepidoptera, extracellular loops 1 and 4 (ECL1 and ECL4) have been shown to be important for selective binding^37^. The importance of ECL1 to binding was demonstrated by a single amino acid polymorphism (Q^125^ vs. E^125^) identified to be responsible for differential toxicity of Cry1Ac against *Spodoptera litura* (highly sensitive) compared to *Spodopter*a *frugiperda* (less sensitive) where the sequence alignment of amino acids between two ABCC2 proteins have 97% identity^59^. Greater sequence homology is observed in ECL1 and ECL4 between CtABCB1 and DvABCB1 than between the loops of either of them when compared to those of DvABCB2 (Supplementary Fig. 1c).

Single nucleotide polymorphism (SNP) markers identified in a single autosomal linkage group (LG8, 115–135 cM) were found to be correlated with resistance to Cry3Bb1 in field populations of WCR^49^. Interestingly, one of those markers, CRW424 (at 119.6 cM), corresponds to *DvABCB2* while the other marker (CRW918) was identified as a cytochrome *P450* gene^49^. Although the linkage of these genes to Cry3Bb1 resistance was strong, the causal gene for Cry3Bb1 resistance has not been confirmed and has yet to be reported. In *Tribolium casteneum*, TcABCB-3B and TcABCB-3A, the equivalents of DvABCB1 and DvABCB2, respectively^46^ (Supplementary Fig. 1d), have been found within the same linkage group (LG3) separated by about 16 Mb^44^. It would be interesting to understand whether *DvABCB1* is in proximity to *DvABCB2* at the 115–135 cM region on LG8 in WCR when the full genome sequence becomes available. It cannot be ruled out that other receptor candidate genes or mechanisms could be involved in Cry3Bb1 field resistance.

Our gain- and loss-of-function results clearly demonstrate a role of DvABCB1 in Cry3A toxicity to WCR. Previous work with mCry3A-resistant larvae showed high resistance in diet bioassay to mCry3A toxin, as well as reduced binding in mCry3A-resistant midgut tissue (21). Here we report decreased expression of *DvABCB1* measured by qRT-PCR and the identification of splice variants in mCry3A-resistant WCR *DvABCB1* sequences (*DvABCB1_3AR1* and *DvABCB1_3AR2*) that can explain the resistance and reduced binding. The presence of splice variants in receptor sequences from resistant insects has been reported, including mis-splicing of *ABCC2* and cadherin-like genes in pink bollworm (*Pectinophora gossypiella*), as well as in other insect species. Similarly, alternatively spliced genes have been identified in resistance mechanisms to non-protein insecticides also, indicating that this mechanism of resistance is common^60–62^.

The large sequence deletions in *DvABCB1* likely alter protein structure and functionality. Both *3AR1* and *3AR2* include deletions that would eliminate the predicted transmembrane helix 6 (TM6) sequence and the loss of most of the first nucleotide binding domain (NBD1). In addition to the loss of TM6, the other 810 amino acids missing from *3AR2* would eliminate the entire large intracellular loop with NBD1, TM7 through TM12 and the corresponding extracellular loops, as well as NBD2. Both NBD are needed to have a functional transporter, thus the deletions result in loss of transport function for both 3AR1 and 3AR2 putative proteins. The lack of TM6 would result in the first large normally cytoplasmic loop in 3AR1 and the entire C-terminus in 3AR2 to be extracellular, if expressed. ECL4 that has been implicated in binding of Cry toxins for other ABC transporters would be located intracellularly or absent in 3AR1 or 3AR2, respectively. Regardless, the extent of the protein deletions would impact protein folding such that the protein would likely be severely structurally altered or never reach the plasma membrane. The result in either scenario would be loss of binding and functionality as the Cry3A receptor in mCry3A-resistant WCR.

Overall, our findings clearly establish a functional role for DvABCB1 in Cry3 toxicity and the alternatively-spliced variants of the *DvABCB1* transcript in addition to the overall decreased expression in mCry3A-resistant WCR reveal a mechanism through which resistance to Cry3A can occur. Since cross-resistance among Cry3 toxins has been demonstrated in both field and lab populations^11,15,16^, DvABCB1 may be the common Cry3 toxin receptor.

## Materials and methods

### Production of double-stranded RNA by in vitro transcription

DNA fragment of 155 base pair regions of *DvABCB1* or *DvABCB2* cDNA sequence was amplified by overlapping extension PCR using four complementary DNA oligodeoxyribonucleotide (oligo) primers^63^. The gene-specific primers also contained promoter sites for T7 RNA polymerase at external oligos for overlapping extension (Table 1). The PCR product served as the template for dsRNA synthesis by in vitro transcription (IVT) using MEGAscript kit (Life Technologies, Carlsbad, CA). IVT products were purified by MEGAclear Kit and quantified by NanoDrop 8,000 (Life Technologies). *GUS* GenBank accession no. S69414.1.

**Table 1:** Oligo Sequences for dsRNA Synthesis by IVT.

Name	Oligo designation	Sequence
*DvABCB1*	5′ external forward	TAATACGACTCACTATAGGGTGACAGAAGAAAAAAAACATAGTATAAAGGATAAAGAGAA
	Internal reverse	TCCTTTGGTTCTTCACTATTAACAAATTGGGCATCAATACCAATTTTCTCTTTATCCTTT
	Internal forward	TGAAGAACCAAAGGAAAAAATTAAGAATGTATCTTTTCCTCAGATGTTTAGGTATGCAAG
	3′ external reverse	TAATACGACTCACTATAGGGTACCATTAAAAATTTATCATAAGTACTTGCATACCTAAAC
*DvABCB2*	5′ external forward	TAATACGACTCACTATAGGGAACTCTATCGGCAGTAATCACTGGTTGTCTACCACCAATA
	Internal reverse	ATATTGCACAGCATTTCCTGCAAGTTCTCCGAATAAAATTGTATTTATTGGTGGTAGACA
	Internal forward	AATGCTGTGCAATATGCTGAAACTTTATATAACGCTACCTTATCACAAAACGAACAAGCT
	3′ external reverse	TAATACGACTCACTATAGGGCATCAAAGAATTTTTCCTGAGCCTCAGCTTGTTCGTTTTG
*GUS*	5′ external forward	ATGTTACGTCCTGTAGAAACCCCAACCCGTGAAATCAAAA
	Internal reverse	CCACAGTTTTCGCGATCCAGACTGAATGCCCACAGGCCGTCGAGTTTTTTGATTTCACGG
	Internal forward	ATCGCGAAAACTGTGGAATTGATCAGCGTTGGTGGGAAAGCGCGTTACAAGAAAGCCGGG
	3′ external reverse	TTAAAACTGCCTGGCACAGCAATTGCCCGGCTTTCTTGTA

### Quantitative real-time PCR (qRT-PCR)

The expression of *DvABCB1* or *DvABCB2* gene was quantified from WCR larvae after 4-day feeding on diet incorporated with 100 µg/mL *DvABCB1* or *DvABCB2* fragment dsRNA. The designs of primer and probe regions are listed in Table 2. Gene expression was analyzed using one-step real-time quantitative RT-PCR. The assay was run, with 2 replicates per sample, using a single-plex set up with Bioline Sensifast Probe Lo Rox kit (Taunton, MA) and analyzed using the 2^−ΔΔCt^ method based on the relative expression of the target gene and reference gene *DvRPS10* (GenBank accession no. KU756281).

**Table 2:** Primers and probes for qRT-PCR assays.

Primers and probes	Sequence
*DvABCB1* F	TCTTACAACCCCTAAATACGATTCTC
*DvABCB1* R	AGGGCAAAATATCGGATACCATC
*DvABCB1* P (FAM)	TGCAATGATATCTCCTGTGAGGCTACCA
*DvABCB2 F*	AGCAATACCAGCATCCTTGG
*DvABCB2 R*	CACCAAAAGCTGTTACCGTTC
*DvABCB2 P (FAM)*	AAAACGAAATGGAAGCCTACGCAGC
*DvRPS1010* F	CTAACTCTGGCATCGAATACCTC
*DvRPS10* R	TGGGCGTTTCAAGGTAGATG
*DvRPS10* (TET)	TTCTCCAGGTAAGTGTAAGAATGTGCGG

### Cell-based assays of DvABCB1

For Sf9 cell assay, *DvABCB1* was amplified by PCR from WCR cDNA (WCRABCB1For2_5′-GGATCCATGACAGAAGAAAAAAAACATAGTATAAAGG-3′ WCRABCB1Rev2_5′-CTCGAGTTAGTGATGGTGATGGTGGTGCGTTTTTTGAGTATATAATTTGTAGTACAGTC-3′) and product was ligated into BluntII-TOPO. The insert was sequence verified and cloned into the pBacPAK9 transfer vector (Clontech Laboratories, Inc. A Takara Bio Company, 1290 Terra Bella Ave, Mountain View, CA 94043) with a 6 × C-terminal His-tag using Gibson assembly (ABCB1GibF_5′-TAAAAAAACCTATAAATACGATGACAGAAGAAAAAAAACAC-3′, ABCB1GibR_5′-TACCGAGCTCGAATTCCCGGTTAGTGATGGTGATGGTG-3′) by NEBuilder HiFi DNA Assembly Cloning Kit (New England Biolabs, Ipswich, MA 01938) and sequence verified. Eleven silent single nucleotide polymorphisms SNPs (nt position 21: T to C; 1329: G to A; 1647: T to C; 1654: T to C; 1657: T to C; 1659: A to G; 1671: G to A; 1761: A to C; 1869: T to C; 2055: G to A; 3288: C to A) and one nonsynonymous SNP (nt position 1501: T to G, resulting in an F to V change) were found in the PCR clone. Transfection of Sf9 cells and production of recombinant baculovirus followed the recommendations by the manufacturer (Clontech Laboratories) for BacPAK Baculovirus Expression System with the following modification: cotransfection was done in 12-well plate (single well seeded at 5 × 10^5^ cells/well) and 0.5X Bacfectin-DNA mixture used for the generation of recombinant virus. For cell assay, Sf9 suspension cells were grown in 15 mL shaker flask at 1 × 10^6^ cells/mL and infected with P2 stock at M.O.I. (multiplicity of infection) of 10. The following day the cells were seeded into a 24-well plate and allowed to attach. The medium was replaced with medium containing individual toxins (trypsin activated Cry3Aa, chemotrypsin activated Cry34Ab1/35Ab1, or Cry6Aa full length protein) at 1 µg/mL. After overnight incubation, toxic effects or cell death was imaged under microscope (Leica DMI4000 inverted microscope equipped with a Leica DFC7000T camera).

For HEK293 cell assay, the DvABCB1 sequence was codon-optimized for HEK293 cell expression by adding a 5′ Kozak sequence and then synthesized (GenScript, Piscataway, NJ). A FLAG tag (MDYKDDDDK) and TagRFP (GenBank no ARG42305) encoding sequences were added to the N- or C-terminus, respectively, and the entire codon-optimized sequence was cloned into the HindIII and XhoI sites of pCDNA3.1 vector.

HEK293 cells were transfected with the DvABCB1 plasmid using Lipofectamine 3000 transfection reagent and selected with 1 mg/mL of Geneticin for 4 weeks. Expression of DvABCB1-RFP fusion protein was shown as an indicator of DvABCB1 positive cells. The cells were examined under confocal microscope equipped with fluorescence detection (Leica TCS SPE, Leica Microsystems, Mannheim, Germany). Cells were seeded in 96-well plates overnight before testing for response to IP3-H9 (0.01–100 nM). Exposure to toxin was carried out for 24 h before cell viability was evaluated using CellTiter-Glo Viability assays following manufacturer’s instruction (Promega, Madison, WI). The luminescence of 10 μM oligomycin challenged cells was subtracted from that of the toxin treated cells as the background absorbance.

### RNAi of *DvABCB1* in WCR using diet bioassay

Non-diapausing western corn rootworm (*D. virgifera virgifera*, WCR) eggs were obtained from Corteva Agriscience insectary (Johnston, IA). The WCR eggs were incubated at 25 °C for 10–12 days prior to processing for egg hatching. Eggs were washed from the oviposition dishes with DI water, surface sterilized with 70% ETOH for 3 min and triple rinsed with sterile DI water. The eggs were resuspended in 0.08% water agar containing 1% (vol./vol.) formaldehyde. The egg-water agar suspension was then spread in a petri dish (150 mm × 25 mm) that contained a 2% water-agar bed and a double layer of filter paper for hatching. Upon hatching, the larvae were transferred to Corteva proprietary diet and allowed to feed for < 24 h prior use.

A 2-step diet bioassay method as described in^27^ was used with some modification. All assays were performed in 96-well assay plates following a diet-incorporation method. Test samples (dsRNA, IP3-H9, IPD072Aa, water or buffer) were mixed with the diet and allowed to cool at room temperature prior infesting with WCR larvae.

### Step-1: Exposure of WCR neonates to dsRNA

Step-1 involved exposure of WCR neonates to dsRNA for 4 days. Wells of 96-well plates (Costar 96 well “U” bottom Plate, Corning Incorporated, NY, USA) were filled with 5 µL of *DvABCB1* dsRNA, *GUS* dsRNA, or water and 25 µL of WCR larval diet and mixed well to homogeneity. The final dsRNA concentrations were 100 ng/µL dsRNA. A total of 2 plates for each of *DvABCB1* dsRNA and *GUS* dsRNA and 4 plates for water treatment were arranged. Prepared plates were infested with < 24 h diet fed neonates (two larvae/well), sealed with vented Mylar and incubated at 27 ± 1 °C and 30 ± 5% relative humidity for 48 h. After 48 h, all surviving larvae were transferred (2 larva/well) to a new plate containing 100 ng/µL *DvABCB1* dsRNA, *GUS* dsRNA or water prepared as described above and assay plates were incubated for additional 48 h. At the end of step 1 exposure (4 days total exposure time), six live WCR larvae were collected individually from each treatment, flash frozen in liquid nitrogen and stored in − 80 °C for qRT-PCR.

### Step 2: Exposure to target toxins

Step 2 involved exposure of larvae from step 1 to the test treatment combinations (Table 1). For these treatments, 25 µL of the desired sample working stock, buffer, or water was mixed with 75 µL of diet in each well of the 96-well assay plate (NUNC plates,). Thirty-two randomly picked 4 day old larvae from each step-1 treatment were exposed to diet that contained IP3-H9 or IPD072Aa (both at 200 ng/µL), water, or buffer (1 × PBS) as control (Table 3). Prepared assay plates were incubated at 27 ± 1 °C and 30 ± 5% relative humidity for 10 days. At the end of step 2 exposure (10 days), assay plates were scored for insect mortality and severe growth inhibition^20,21,47^. Assays were performed with four replicates per treatment (n = 8 larvae per replicate and n = 32 per treatment). The entire experiment was performed twice (n = 96 per treatment).

**Table 3:** Exposure of *D. virgifera virgifera* to dsRNA and insecticidal proteins following a 2-step exposure bioassay method.

Treatment in Step-1 (4 days exposure)	Treatment in Step-2 (10 days exposure)	Treatment type
*DvABCB1*^a^	IP3-H9	Test sample
*DvABCB1*	IPD072Aa	Test sample
*DvABCB1*^a^	Water	Negative control
*DvABCB2*^a^	IP3-H9	Test sample
*DvABCB2*^a^	Water	Negative control
*GUS*^a^	IP3-H9	Test sample
*GUS*	IPD072Aa	Test sample
*GUS*^a^	Water	Negative control
WATER^a^	IP3-H9	Positive control
WATER	IPD072Aa	Positive control
WATER^a^	Buffer (1 × PBS)	Negative control

Step-1 exposure to dsRNA at 100 µg/mL for 4 days and step-2 exposure to test toxin samples at 200 ng/µl for 10 days. Total assay duration was 14 days. Studies for effects on IPD072Aa and IP3-H9 toxicity were performed with their own positive and negative control set. A separate study was performed to compare the impact of silencing *DvABCB1* or *DvABCB2* on IP3-H9 toxicity.

^a^Samples included in the second set of functional bioassays for IP3-H9 only.

For IP3-H9, additional functional bioassay was performed to investigate whether silencing of *DvABCB2* impacts the activity of IP3-H9 in similar manner as *DvABCB1*. The assay was setup following similar procedures as described above and the list of treatments are indicated in Table 3. There were four replicates per treatment (n = 8 larvae per replicate and n = 32 per treatment). The entire experiment was replicated twice (n = 64 per treatment).

### Data analysis

Data analysis was performed using SAS Enterprise guide v.8.1^64^. Proportional mortality (number dead/total exposed) and growth inhibition (dead plus severely stunted larvae/total exposed) data were subjected to statistical analysis using general linear mixed model with a binomial distribution, a logit link function, and the Laplace likelihood approximation method. For the second set of bioassays, because of several zero values for the negative controls, the actual percent mortality data was used for analysis and in such cases the Gaussian distribution and identity link function with the Laplace likelihood approximation method was used. In all cases, treatment was considered a fixed effect and replicates within treatments in each experiment were treated as a random effect. Least square means pairwise comparisons between treatments with Tukey’s multiplicity adjustments were performed at alpha level of 0.05. Means are presented as percent and the error bars reported using the 95% confidence limits.

Relative gene expression data was subjected to statistical analysis using a mixed model. Treatment was considered a fixed effect and replicate PCR samples within an insect in each treatment were treated as a random effect. Least square means pairwise comparisons between treatments with Tukey’s multiplicity adjustments were performed at alpha level of 0.05. Bar represented mean ± SEM.

## Supplementary information

Supplementary Information.

## Data Availability

DvABCB1 and DvABCB2 sequences have been deposited in the GenBank of National Center for Biotechnology Information under the accession numbers MN908590 and MN908591.
