# Supplementary material for: Functional validation of DvABCB1 as a receptor of Cry3 toxins in western corn rootworm, Diabrotica virgifera virgifera
Source: Sci Rep. 2020 Sep 28;10:15830. doi: 10.1038/s41598-020-72572-9 (PMC7522262; doi:10.1038/s41598-020-72572-9)
Supplement: Supplementary file 1 — Supplementary Information. [file 41598_2020_72572_MOESM1_ESM.docx]

***Supplementary Information for***

Functional validation of DvABCB1 as a receptor of Cry3 toxins in western corn rootworm, *Diabrotica virgifera virgifera*

Xiping Niu^1*^, Adane Kassa^1^, James Hasler^1,2^, Samantha Griffin^3^, Claudia Perez-Ortega^4^, Lisa Procyk^1^, Jun Zhang^1^, Deirdre M. Kapka-Kitzman^1^, Mark E. Nelson^1*^, and Albert Lu^1^

^1^Corteva Agriscience, 7300 NW 62^nd^ Ave., Johnston, IA, 50131, USA

^2^present address: 1628 S CR250 W, Danville, IN, 46122

^3^present address: Corteva Agriscience, 9330 Zionsville Rd, Indianapolis, IN 46268

^4^present address: Reaction Biology Corp, 1 Great Valley Pkwy Ste 2, Malvern, PA. 19355

^*^Corresponding Author:

Xiping Niu, Telephone: 515 535-3605, E-mail: xiping.niu[@corteva.com](mailto:xu.hu@pioneer.com)

Or

Mark Nelson, Telephone: 515 535-6845, E-mail: mark.e.nelson@corteva.com

**Materials and Methods**

**Transcriptome assembly, RNA-seq and protein analyses.**

RNA sample preparation, transcriptome assembly, and RNA-seq were as previously described (1). Briefly, cDNA prepared from 14 WCR life stages and dissected tissues (eggs, neonates, midguts of third instar larvae, and testes and ovaries from male and female adults) were sequenced by Illumina and 454 Titanium sequencing technologies. *De novo* transcriptome assemblies were performed using Trinity (v. 2.0.6), IDBA-Tran (v. 1.1.1), Velvet-Oases (v. 1.2.10–0.2.08), and/or SOAPdenovo-Trans (v. 1.03) (2-5). WCR DvABCB1 and DvABCB2 were identified by blast searches using leaf beetle CtABCB1 (6). DvABCB1 protein domains were predicted by InterProScan (7). Percent identity scores were calculated from single pairwise alignments using AlignX from Vector NTI Advance version 9.1.0 (Invitrogen). Alignments among CtABCB1, DvABCB1, and DvABCB1 were done by ClustraW using Geneious 10.2.6 software package (8). Phylogenetic tree was constructed using Geneious Tree Builder by pairwise alignment using Blosum62 matrix and the genetic distance calculated by Neighbor-Joining method using Jukes-Cantor model.

***DvABCB1* sequence determination in susceptible and mCry3A-resistant WCR.**

Total RNA was isolated from frozen guts that were extracted from susceptible and mCry3A-resistant 3^rd^ instar insects (in groups of 10 guts) following the RNeasy protocol with optional on-column DNase digestion (Qiagen, Hilden, Germany). To obtain amplicons for sequencing, RT-PCR was performed using cDNA generated with SuperScriptIII reverse transcriptase (Life Technologies, Carlsbad, CA). PCR reactions for *DvABCB1* were setup using Invitrogen Platinum SuperFi PCR Master Mix (Life Technologies, Carlsbad, CA) using cDNA from each strain (DvABCB1FwdStrt_5’-ATGACAGAAGAAAAAAAACATAGTATAAAGG-3’ and DvABCB1RevStop_5’-TTACGTTTTTTGAGTATATAATTTGTAGTACAG-3’). The resulting PCR amplicons were purified and concentrated using Ampure PB beads prior to library preparation.  Using Pacific Bioscience’s Amplicon Barcoding kit, uniquely barcoded adapters were ligated to each amplicon.  Following pooling the library was bound to a sequencing primer and polymerase using the Sequel Binding Kit 3.0.  This was loaded onto a single cell on the Sequel with a run configuration of 20 hours using the Sequel Sequencing Kit 3.0. CCS reads were generated using the ccs application (version 4.0.0, Pacific Biosciences, Inc.) with minPasses = 3 and minPredictedAccuracy = 0.999.  Sequences that began with the correct unique barcode and primer sequence were extracted and split into their own FASTA files.  Within each sample, total percentage of molecules were calculated for amplicon sequences. Sequences with occurrences of at least 5% of total molecules were analyzed. The 5% threshold helps to eliminate reads that have low frequency errors from PCR amplification or sequencing.

**References**

1. Davis-Vogel C, et al. (2018) Identification and comparison of key RNA interference machinery from western corn rootworm, fall armyworm, and southern green stink bug. *PLoS One* 13(9):e0203160.

2. Peng Y, Leung HC, Yiu SM, & Chin FY (2012) IDBA-UD: a *de novo* assembler for single-cell and metagenomic sequencing data with highly uneven depth. *Bioinformatics* 28(11):1420-1428.

3. Xie Y, et al. (2014) SOAPdenovo-Trans: *de novo* transcriptome assembly with short RNA-Seq reads. *Bioinformatics* 30(12):1660-1666.

4. Schulz MH, Zerbino DR, Vingron M, & Birney E (2012) Oases: robust *de novo* RNA-seq assembly across the dynamic range of expression levels. *Bioinformatics* 28(8):1086-1092.

5. Grabherr MG, et al. (2011) Full-length transcriptome assembly from RNA-Seq data without a reference genome. *Nat Biotechnol* 29(7):644-652.

6. Pauchet Y, Bretschneider A, Augustin S, & Heckel DG (2016) A P-glycoprotein is linked to resistance to the *Bacillus thuringiensis* Cry3Aa toxin in a leaf beetle. *Toxins (Basel)* 8(12).

7. Apweiler R, et al. (2001) The InterPro database, an integrated documentation resource for protein families, domains and functional sites. *Nucleic Acids Res* 29(1):37-40.

8. Kearse M, et al. (2012) Geneious Basic: an integrated and extendable desktop software platform for the organization and analysis of sequence data. *Bioinformatics* 28(12):1647-1649.

**Supplementary Table and Figures**

**Supplementary Table 1. Potency of IP3-H9 and IPD072Aa against diet fed 4-day old WCR larvae.**

**Supplementary Figures**

**Supplementary Figure 1. Predicted structure of DvABCB1 and protein sequence alignment of DvABCB1, DvABCB2, and CtABCB1**. (a) Graphic representation of predicted DvABCB1 structural features: Transmembrane helix (TM) (yellow diamond) from TM1-6 to TM7-12 in each TMD followed by NBD (red rectangle) in each half of DvABCB1 transporter. ECL regions (1-6) are indicated on the top between TMs. (b) Protein sequence alignment among CtABCB1, DvABCB1, and DvABCB2 showing TM (box in red), NBD (box in black) and ECL regions. The alignment was done by ClustraW using Blosum62 score matrix in Geneious 10.2.6. The identity of residues highlighted: Green: 100%; Yellow: 60-80%; Not highlighted: below 60%. (c) Alignments and percent identities of ECLs 1 and 4 of DvABCB1, DvABCB2, and CtABCB1. The alignment and identity calculation were done by ClustraW using Blosum62 score matrix in Geneious 10.2.6. The identity of residues highlighted is as in *B*. (d) Phylogenetic tree of coleopteran ABCB proteins in GenBank. The tree is drawn to scale, with branch lengths in the same units as those of the evolutionary distances used to infer the phylogenetic tree. CtABCB1 and DvABCB1 are indicated in a dashed rectangular square. Coleopteran ABCB family members with GenBank accession nos. as follows: *Diabrotica virgifera* (DvABCB1, MN908590; DvABCB2, MN908591), *Chrysomela tremulae* (CtABCB1, GU462154), *Leptinotarsa decemlineata* (LdMRP1B-L: multidrug resistance protein 1B-like, XP_023021695), and *Tribolium castaneum* (TcABCB-3A, XP_967244.2; TcABCB-3B, XP_001810982.1; TcABCB-4A, XP_966724.1; TcABCB-5A, XP_001813375.1).

**Supplementary Figure 2. Specificity of dsRNA targeting *DvABCB1* and *DvABCB2* in *D. virgifera virgifera* larvae after dsRNA treatments.** qRT-PCR assays were done on WCR larvae 4 days after dsRNA treatments (water, *GUS*, *DvABCB1* and *DvABCB2*). Relative expression of *DvABCB1 or DvABCB2* by qRT-PCR assay is shown for each treatment using *DvRPS10* as a reference and normalized to *DvABCB1 or DvABCB2* expression in water control (mean ± SE; n = 14-16 per treatment (7-8 insects per treatment and n=2 PCR sub samples per insect)). (a) *DvABCB1* expression (F _(3, 58)_ = 7.25, *p*-value < 0.001. (b) *DvABCB2* expression (F _(3, 60)_ = 12.88, *p*-value < 0.001. Least square means pairwise comparison *p*-values: > 0.05 ns (not significant), < 0.001***.

Consensus ATGACAGAAG AAAAAAAACA TAGTATAAAG GATAAAGAGA AAATTGGTAT TGATGCCCAA 60

DvABCB1_FL_Sus ATGACAGAAG AAAAAAAACA TAGTATAAAG GATAAAGAGA AAATTGGTAT TGATGCCCAA 60

DvABCB1_3AR1 ATGACAGAAG AAAAAAAACA TAGTATAAAG GATAAAGAGA AAATTGGTAT TGATGCCCAA 60

DvABCB1_3AR2 ATGACAGAAG AAAAAAAACA TAGTATAAAG GATAAAGAGA AAATTGGTAT TGATGCCCAA 60

DvABCB1_3AR3 ATGACAGAAG AAAAAAAACA TAGTATAAAG GATAAAGAGA AAATTGGTAT TGATGCCCAA 60

Consensus TTTGTTAATA GTGAAGAACC AAAGGAAAAA ATTAAGAATG TATCTTTTCC TCAGATGTTT 120

DvABCB1_FL_Sus TTTGTTAATA GTGAAGAACC AAAGGAAAAA ATTAAGAATG TATCTTTTCC TCAGATGTTT 120

DvABCB1_3AR1 TTTGTTAATA GTGAAGAACC AAAGGAAAAA ATTAAGAATG TATCTTTTCC TCAGATGTTT 120

DvABCB1_3AR2 TTTGTTAATA GTGAAGAACC AAAGGAAAAA ATTAAGAATG TATCTTTTCC TCAGATGTTT 120

DvABCB1_3AR3 TTTGTTAATA GTGAAGAACC AAAGGAAAAA ATTAAGAATG TATCTTTTCC TCAGATGTTT 120

Consensus AGGTATGCAA GTACTTATGA TAAATTTTTA ATGGTAGTAG GACTTATATC GGCAACTGGA 180

DvABCB1_FL_Sus AGGTATGCAA GTACTTATGA TAAATTTTTA ATGGTAGTAG GACTTATATC GGCAACTGGA 180

DvABCB1_3AR1 AGGTATGCAA GTACTTATGA TAAATTTTTA ATGGTAGTAG GACTTATATC GGCAACTGGA 180

DvABCB1_3AR2 AGGTATGCAA GTACTTATGA TAAATTTTTA ATGGTAGTAG GACTTATATC GGCAACTGGA 180

DvABCB1_3AR3 AGGTATGCAA GTACTTATGA TAAATTTTTA ATGGTAGTAG GACTTATATC GGCAACTGGA 180

Consensus ACAGGAGTCT TACAACCCCT AAATACGATT CTCTTTGGTA GCCTCACAGG AGATATCATT 240

DvABCB1_FL_Sus ACAGGAGTCT TACAACCCCT AAATACGATT CTCTTTGGTA GCCTCACAGG AGATATCATT 240

DvABCB1_3AR1 ACAGGAGTCT TACAACCCCT AAATACGATT CTCTTTGGTA GCCTCACAGG AGATATCATT 240

DvABCB1_3AR2 ACAGGAGTCT TACAACCCCT AAATACGATT CTCTTTGGTA GCCTCACAGG AGATATCATT 240

DvABCB1_3AR3 ACAGGAGTCT TACAACCCCT AAATACGATT CTCTTTGGTA GCCTCACAGG AGATATCATT 240

Consensus GCATATGCTA CGTCGATACA GATAAATTTA CCTGCCGATC AGAAGAAAAT AGCAGAAGAC 300

DvABCB1_FL_Sus GCATATGCTA CGTCGATACA GATAAATTTA CCTGCCGATC AGAAGAAAAT AGCAGAAGAC 300

DvABCB1_3AR1 GCATATGCTA CGTCGATACA GATAAATTTA CCTGCCGATC AGAAGAAAAT AGCAGAAGAC 300

DvABCB1_3AR2 GCATATGCTA CGTCGATACA GATAAATTTA CCTGCCGATC AGAAGAAAAT AGCAGAAGAC 300

DvABCB1_3AR3 GCATATGCTA CGTCGATACA GATAAATTTA CCTGCCGATC AGAAGAAAAT AGCAGAAGAC 300

Consensus AATTTTTTTG ATGGTATCCG ATATTTTGCC CTAATGAATT CTCTGATCGG AATAGGAATG 360

DvABCB1_FL_Sus AATTTTTTTG ATGGTATCCG ATATTTTGCC CTAATGAATT CTCTGATCGG AATAGGAATG 360

DvABCB1_3AR1 AATTTTTTTG ATGGTATCCG ATATTTTGCC CTAATGAATT CTCTGATCGG AATAGGAATG 360

DvABCB1_3AR2 AATTTTTTTG ATGGTATCCG ATATTTTGCC CTAATGAATT CTCTGATCGG AATAGGAATG 360

DvABCB1_3AR3 AATTTTTTTG ATGGTATCCG ATATTTTGCC CTAATGAATT CTCTGATCGG AATAGGAATG 360

Consensus TTTGTGTTCA GTTACCTAGC AACAGTCACT TTTAACTATT CTGCTATGAG ACAGATATTC 420

DvABCB1_FL_Sus TTTGTGTTCA GTTACCTAGC AACAGTCACT TTTAACTATT CTGCTATGAG ACAGATATTC 420

DvABCB1_3AR1 TTTGTGTTCA GTTACCTAGC AACAGTCACT TTTAACTATT CTGCTATGAG ACAGATATTC 420

DvABCB1_3AR2 TTTGTGTTCA GTTACCTAGC AACAGTCACT TTTAACTATT CTGCTATGAG ACAGATATTC 420

DvABCB1_3AR3 TTTGTGTTCA GTTACCTAGC AACAGTCACT TTTAACTATT CTGCTATGAG ACAGATATTC 420

Consensus AAAATAAGGT CAGCATATTT GAAATCCATT TTAAACCAAG ATGTAGGTTG GTACGATATT 480

DvABCB1_FL_Sus AAAATAAGGT CAGCATATTT GAAATCCATT TTAAACCAAG ATGTAGGTTG GTACGATATT 480

DvABCB1_3AR1 AAAATAAGGT CAGCATATTT GAAATCCATT TTAAACCAAG ATGTAGGTTG GTACGATATT 480

DvABCB1_3AR2 AAAATAAGGT CAGCATATTT GAAATCCATT TTAAACCAAG ATGTAGGTTG GTACGATATT 480

DvABCB1_3AR3 AAAATAAGGT CAGCATATTT GAAATCCATT TTAAACCAAG ATGTAGGTTG GTACGATATT 480

Consensus AATCAGACTG GAGATTTTGC CAGTAGGATG TCAGATGATT TGTTCAAATT TGAAGATGGC 540

DvABCB1_FL_Sus AATCAGACTG GAGATTTTGC CAGTAGGATG TCAGATGATT TGTTCAAATT TGAAGATGGC 540

DvABCB1_3AR1 AATCAGACTG GAGATTTTGC CAGTAGGATG TCAGATGATT TGTTCAAATT TGAAGATGGC 540

DvABCB1_3AR2 AATCAGACTG GAGATTTTGC CAGTAGGATG TCAGATGATT TGTTCAAATT TGAAGATGGC 540

DvABCB1_3AR3 AATCAGACTG GAGATTTTGC CAGTAGGATG TCAGATGATT TGTTCAAATT TGAAGATGGC 540

Consensus ATAGGTGAAA AAGTACCGGT ATTCTGGAGT TTTCAAGTTG TTTTTTTAAC TTCACTTATC 600

DvABCB1_FL_Sus ATAGGTGAAA AAGTACCGGT ATTCTGGAGT TTTCAAGTTG TTTTTTTAAC TTCACTTATC 600

DvABCB1_3AR1 ATAGGTGAAA AAGTACCGGT ATTCTGGAGT TTTCAAGTTG TTTTTTTAAC TTCACTTATC 600

DvABCB1_3AR2 ATAGGTGAAA AAGTACCGGT ATTCTGGAGT TTTCAAGTTG TTTTTTTAAC TTCACTTATC 600

DvABCB1_3AR3 ATAGGTGAAA AAGTACCGGT ATTCTGGAGT TTTCAAGTTG TTTTTTTAAC TTCACTTATC 600

Consensus ATAGCTCTTG TGAAAGGATG GGAACTTGCT TTAATATGTT TAACCTCTCT ACCCGCTACA 660

DvABCB1_FL_Sus ATAGCTCTTG TGAAAGGATG GGAACTTGCT TTAATATGTT TAACCTCTCT ACCCGCTACA 660

DvABCB1_3AR1 ATAGCTCTTG TGAAAGGATG GGAACTTGCT TTAATATGTT TAACCTCTCT ACCCGCTACA 660

DvABCB1_3AR2 ATAGCTCTTG TGAAAGGATG GGAACTTGCT TTAATATGTT TAACCTCTCT ACCCGCTACA 660

DvABCB1_3AR3 ATAGCTCTTG TGAAAGGATG GGAACTTGCT TTAATATGTT TAACCTCTCT ACCCGCTACA 660

Consensus TTAATAACAA TCGGAATTGT AGGACTTCTC ACTACAAAAT TAGCAAAAAA TGAACTGGAA 720

DvABCB1_FL_Sus TTAATAACAA TCGGAATTGT AGGACTTCTC ACTACAAAAT TAGCAAAAAA TGAACTGGAA 720

DvABCB1_3AR1 TTAATAACAA TCGGAATTGT AGGACTTCTC ACTACAAAAT TAGCAAAAAA TGAACTGGAA 720

DvABCB1_3AR2 TTAATAACAA TCGGAATTGT AGGACTTCTC ACTACAAAAT TAGCAAAAAA TGAACTGGAA 720

DvABCB1_3AR3 TTAATAACAA TCGGAATTGT AGGACTTCTC ACTACAAAAT TAGCAAAAAA TGAACTGGAA 720

Consensus GCATATGGCG CTGCTGGATC GATAGCCGAA GAAGCATTGT CTTTAATTAG AACCATAACG 780

DvABCB1_FL_Sus GCATATGGCG CTGCTGGATC AATAGCCGAA GAAGCATTGT CTTTAATTAG AACCATAACG 780

DvABCB1_3AR1 GCATATGGCG CTGCTGGATC GATAGCCGAA GAAGCATTGT CTTTAATTAG AACCATAACG 780

DvABCB1_3AR2 GCATATGGCG CTGCTGGATC GATAGCCGAA GAAGCATTGT CTTTAATTAG AACCATAACG 780

DvABCB1_3AR3 GCATATGGCG CTGCTGGATC GATAGCCGAA GAAGCATTGT CTTTAATTAG AACCATAACG 780

Consensus GCTTTTGGAG GTCAGAAGAA AGAAGTAGAC AGATATAATA AAAATTTAGT TGAAGCCAAA 840

DvABCB1_FL_Sus GCTTTTGGAG GTCAGAAGAA AGAAGTAGAC AGATATAATA AAAATTTAGT TGAAGCCAAA 840

DvABCB1_3AR1 GCTTTTGGAG GTCAGAAGAA AGAAGTAGAC AGATATAATA AAAATTTAGT TGAAGCCAAA 840

DvABCB1_3AR2 GCTTTTGGAG GTCAGAAGAA AGAAGTAGAC AGATATAATA AAAATTTAGT TGAAGCCAAA 840

DvABCB1_3AR3 GCTTTTGGAG GTCAGAAGAA AGAAGTAGAC AGATATAATA AAAATTTAGT TGAAGCCAAA 840

Consensus AACAATAATA TAAGAAGATC GATGTTTTCA GCATTAGGTT TCGGTTTATT GTGGTTCATG 900

DvABCB1_FL_Sus AACAATAATA TAAGAAGATC GATGTTTTCA GCATTAGGTT TCGGTTTATT GTGGTTCATG 900

DvABCB1_3AR1 AACAATAATA TAAGAAGATC GATGTTTTCA GCATTAGGTT TCGGTTTATT GTGGTTCATG 900

DvABCB1_3AR2 AACAATAATA TAAGAAGATC GATGTTTTCA GCATTAGGTT TCGGTTTATT GTGGTTCATG 900

DvABCB1_3AR3 AACAATAATA TAAGAAGATC GATGTTTTCA GCATTAGGTT TCGGTTTATT GTGGTTCATG 900

Consensus ATCTATGCCA GTTATGCTCT GGCATTCTGG TATGGTGTAA AATTAGTTTT AAGGGACAGG 960

DvABCB1_FL_Sus ATCTATGCCA GTTATGCTCT GGCATTCTGG TATGGTGTAA AATTAGTTTT AAGGGACAGG 960

DvABCB1_3AR1 ATCTATGCCA GTTATGCTCT GGCATTCTGG TATGGTGTAA AATTAGTTTT AAGGGACAGG 960

DvABCB1_3AR2 ATCTATGCCA GTTATGCTCT GGCATTCTGG TATGGTGTAA AATTAGTTTT AAGGGACAGG 960

DvABCB1_3AR3 ATCTATGCCA GTTATGCTCT GGCATTCTGG TATGGTGTAA AATTAGTTTT AAGGGACAGG 960

Consensus ACAGCTACGA ATCAAATATA TACTCCTAGT AATATGGTGA CTGNNNNNNN NNNNNNNNNN 1020

DvABCB1_FL_Sus ACAGCTACGA ATCAAATATA TACTCCTAGT AATATGGTGA CTGTTTTCTT TAGTGTCATG 1020

DvABCB1_3AR1 ACAGCTACGA ATCAAATATA TACTCCTAGT AATATGGTGA CTG------- ---------- 1003

DvABCB1_3AR2 ACAGCTACGA ATCAAATATA TACTCCTAGT AATATGGTGA CTG------- ---------- 1003

DvABCB1_3AR3 ACAGCTACGA ATCAAATATA TACTCCTAGT AATATGGTGA CTGTTTTCTT TAGTGTCATG 1020

Consensus NNNNNNNNNN NNNNNNNNNN NNNNNNNNNN NNNNNNNNNN NNNNNNNNNN NNNNNNNNNN 1080

DvABCB1_FL_Sus ACAGGATCTA TGAACTTTGG TATTGCCTCT CCATATATAG AGGCATTTGG AATCTCAAAA 1080

DvABCB1_3AR1 ---------- ---------- ---------- ---------- ---------- ---------- 1003

DvABCB1_3AR2 ---------- ---------- ---------- ---------- ---------- ---------- 1003

DvABCB1_3AR3 ACAGGATCTA TGAACTTTGG TATTGCCTCT CCATATATAG AGGCATTTGG AATCTCAAAA 1080

Consensus NNNNNNNNNN NNNNNNNNNN NNNNNNNNNN NNNNNNNNNN NNNNNNNNNN NNNNNNNNNN 1140

DvABCB1_FL_Sus GCGGCGGCGT CAAAAATTTT TAGTGTAATC GATCACAAAC CTACAATTAA TTTATCAAAA 1140

DvABCB1_3AR1 ---------- ---------- ---------- ---------- ---------- ---------- 1003

DvABCB1_3AR2 ---------- ---------- ---------- ---------- ---------- ---------- 1003

DvABCB1_3AR3 GCGGCGGCGT CAAAAATTTT TAGTGTAATC GATCACAAAC CTACAATTAA TTTATCAAAA 1140

Consensus NNNNNNNNNN NNNNNNNNNN NNNNNNNNNN NNNNNNNNNN NNNNNNNNNN NNNNNNNNNN 1200

DvABCB1_FL_Sus GGAAATGGAA AAACGCTAAA CGTACTCATA GGAAATATAC AATTTAAAGA TGTGGCTTTC 1200

DvABCB1_3AR1 ---------- ---------- ---------- ---------- ---------- ---------- 1003

DvABCB1_3AR2 ---------- ---------- ---------- ---------- ---------- ---------- 1003

DvABCB1_3AR3 GGAAATGGAA AAACGCTAAA CGTACTCATA GGAAATATAC AATTTAAAGA TGTGGCTTTC 1200

Consensus NNNNNNNNNN NNNNNNNNNN NNNNNNNNNN NNNNNNNNNN NNNNNNNNNN NNNNNNNNNN 1260

DvABCB1_FL_Sus CGTTATCCAT CTAGGAAAGA TGTACCGATA TTAAAAGGAT TAAGTTTGAA CATTAAATCT 1260

DvABCB1_3AR1 ---------- ---------- ---------- ---------- ---------- ---------- 1003

DvABCB1_3AR2 ---------- ---------- ---------- ---------- ---------- ---------- 1003

DvABCB1_3AR3 CGTTATCCAT CTAGGAAAGA TGTACCGATA TTAAAAGGAT TAAGTTTGAA CATTAAATCT 1260

Consensus NNNNNNNNNN NNNNNNNNNN NNNNNNNNNN NNNNNNNNNN NNNNNNNNNN NNNNNNNNNN 1320

DvABCB1_FL_Sus GGAGACACTG TGGCACTTGT AGGTAGTTCG GGCTGTGGAA AATCTACGGT TATTCAGTTA 1320

DvABCB1_3AR1 ---------- ---------- ---------- ---------- ---------- ---------- 1003

DvABCB1_3AR2 ---------- ---------- ---------- ---------- ---------- ---------- 1003

DvABCB1_3AR3 GGAGACACTG TGGCACTTGT AGGTAGTTCG GGCTGTGGAA AATCTACGGT TATTCAGTTA 1320

Consensus NNNNNNNN-N NNNNNNNNNN NNNNNNNNNN NNNNNNNNNN NNNNNNNNNN NNNNNNNNNN 1379

DvABCB1_FL_Sus CTTCAACGAT TATACGATGC GGACTCTGGA GAGGTCACCA TAGACGGAAA AAATATTAAA 1380

DvABCB1_3AR1 ---------- ---------- ---------- ---------- ---------- ---------- 1003

DvABCB1_3AR2 ---------- ---------- ---------- ---------- ---------- ---------- 1003

DvABCB1_3AR3 CTTCAACGGT TATACGATGC GGACTCTGGA GAGGTCACCA TAGACGGAAA AAATATTAAA 1380

Consensus NNNNNNNNNN NNNNNNNNNN NNNNNNNNNN NNNNNNNNNN NNNNNNNNNN NNNNNNNNNN 1439

DvABCB1_FL_Sus GAATACGATT TAACTTGGTT AAGAAGCCAA ATAGGAGTTG TAGGTCAAGA ACCAATTCTC 1440

DvABCB1_3AR1 ---------- ---------- ---------- ---------- ---------- ---------- 1003

DvABCB1_3AR2 ---------- ---------- ---------- ---------- ---------- ---------- 1003

DvABCB1_3AR3 GAATACGATT TAACTTGGTT AAGAAGCCAA ATAGGAGTTG TAGGTCAAGA ACCAATTCTC 1440

Consensus NNNNNNNNNN NNNNNNNNNN NNNNNNNNNN NNNNNNNNNN NNNNNNNNNN NNNNNNNNNN 1499

DvABCB1_FL_Sus TTTGGAACAT CCATTTTAGA AAATATTAGG TACGGTAAAG ATGGAGTAAC AGAGGAAGAT 1500

DvABCB1_3AR1 ---------- ---------- ---------- ---------- ---------- ---------- 1003

DvABCB1_3AR2 ---------- ---------- ---------- ---------- ---------- ---------- 1003

DvABCB1_3AR3 TTTGGAACAT CCATTTTAGA AAATATTAGG TACGGTAAAG ATGGAGTAAC AGAGGAAGAT 1500

Consensus NNNNNNNNNN NNNNNNNNNN NNNNNNNNNN NNNNNNNNNN NNNNNNNNNN NNNNNNNNNN 1559

DvABCB1_FL_Sus GTCATTCAAG CAGCTAAAAA AGCTAATGCG CATAACTTTA TTAAAGCTCT TCCTAACGGA 1560

DvABCB1_3AR1 ---------- ---------- ---------- ---------- ---------- ---------- 1003

DvABCB1_3AR2 ---------- ---------- ---------- ---------- ---------- ---------- 1003

DvABCB1_3AR3 GTCATTCAAG CAGCTAAAAA AGCTAATGCG CATAACTTTA TTAAAGCTCT TCCTAACGGA 1560

Consensus NNNNNNNNNN NNNNNNNNNN NNNNNNNNNN NNNNNNNNNN NNNNNNNNNN NNNNNNNNNN 1619

DvABCB1_FL_Sus TATAATACAT TGGTTGGAGA GAAAGGGGCT CAACTCTCAG GTGGACAAAA ACAAAGGATA 1620

DvABCB1_3AR1 ---------- ---------- ---------- ---------- ---------- ---------- 1003

DvABCB1_3AR2 ---------- ---------- ---------- ---------- ---------- ---------- 1003

DvABCB1_3AR3 TATAATACAT TGGTTGGAGA GAAAGGGGCT CAACTCTCAG GTGGACAAAA ACAAAGGATA 1620

Consensus NNNNNNNNNN NNNNNNNNNN NNNNNN-NNN NNN-NN-N-N NNNNNNNNNN -NNNNNNNNN 1674

DvABCB1_FL_Sus GCTATAGCTA GAGCTTTGGT AAGAAACCCT ACTCTACTGC TTTTGGACGA AGCTACTTCA 1680

DvABCB1_3AR1 ---------- ---------- ---------- ---------- ---------- ---------- 1003

DvABCB1_3AR2 ---------- ---------- ---------- ---------- ---------- ---------- 1003

DvABCB1_3AR3 GCTATAGCTA GAGCTTTGGT AAGAAATCCT ACTTTATTAC TTTTGGACGA GGCTACTTCA 1680

Consensus NNNNNNNNNN NNNNNNNNNN NNNNNNNNNN NNNNNNNNNN NNNNNNNNNC AAGTGTTGAA 1734

DvABCB1_FL_Sus GCACTGGATA ATACCAGTGA AGCTAAAGTA CAAGCAGCTC TAGATGCGGC AAGTGTTGAA 1740

DvABCB1_3AR1 ---------- ---------- ---------- ---------- ---------C AAGTGTTGAA 1014

DvABCB1_3AR2 ---------- ---------- ---------- ---------- ---------- ---------- 1003

DvABCB1_3AR3 GCACTGGATA ATACCAGTGA AGCTAAAGTA CAAGCAGCTC TAGATGCGGC AAGTGTTGAA 1740

Consensus TGTACAACTA TAATTGTGGC ACACAGGCTA TCAACAATTC GAGGTGCAAA CAAAATTATC 1794

DvABCB1_FL_Sus TGTACAACTA TAATTGTGGC CCACAGGCTA TCAACAATTC GAGGTGCAAA CAAAATTATC 1800

DvABCB1_3AR1 TGTACAACTA TAATTGTGGC ACACAGGCTA TCAACAATTC GAGGTGCAAA CAAAATTATC 1074

DvABCB1_3AR2 ---------- ---------- ---------- ---------- ---------- ---------- 1003

DvABCB1_3AR3 TGTACAACTA TAATTGTGGC ACACAGGCTA TCAACAATTC GAGGTGCAAA CAAAATTATC 1800

Consensus GTACTTTCTC AAGGAGTCGT GGTAGAAGAA GGAACCCACG AAGAACTAAT GGAACTTAAA 1854

DvABCB1_FL_Sus GTACTTTCTC AAGGAGTCGT GGTAGAAGAA GGAACCCACG AAGAACTAAT GGAACTTAAA 1860

DvABCB1_3AR1 GTACTTTCTC AAGGAGTCGT GGTAGAAGAA GGAACCCACG AAGAACTAAT GGAACTTAAA 1134

DvABCB1_3AR2 ---------- ---------- ---------- ---------- ---------- ---------- 1003

DvABCB1_3AR3 GTACTTTCTC AAGGAGTCGT GGTAGAAGAA GGAACCCACG AAGAACTAAT GGAACTTAAA 1860

Consensus CAAGAGTATT ATAGATTAGT AACAGCCCAA GTCAAATCAT CGGAACAGTT TGAAGTCGCT 1914

DvABCB1_FL_Sus CAAGAGTACT ATAGATTAGT AACAGCCCAA GTCAAATCAT CGGAACAGTT TGAAGTCGCT 1920

DvABCB1_3AR1 CAAGAGTATT ATAGATTAGT AACAGCCCAA GTCAAATCAT CGGAACAGTT TGAAGTCGCT 1194

DvABCB1_3AR2 ---------- ---------- ---------- ---------- ---------- ---------- 1003

DvABCB1_3AR3 CAAGAGTATT ATAGATTAGT AACAGCCCAA GTCAAATCAT CGGAACAGTT TGAAGTCGCT 1920

Consensus GAAAAGAAGA AAGTGGTAAG AGCTATTAGC TTGGCAGAAA GTAGCACAGG CTCTGACCAT 1974

DvABCB1_FL_Sus GAAAAGAAGA AAGTGGTAAG AGCTATTAGC TTGGCAGAAA GTAGCACAGG CTCTGACCAT 1980

DvABCB1_3AR1 GAAAAGAAGA AAGTGGTAAG AGCTATTAGC TTGGCAGAAA GTAGCACAGG CTCTGACCAT 1254

DvABCB1_3AR2 ---------- ---------- ---------- ---------- ---------- ---------- 1003

DvABCB1_3AR3 GAAAAGAAGA AAGTGGTAAG AGCTATTAGC TTGGCAGAAA GTAGCACAGG CTCTGACCAT 1980

Consensus AACATAGAGG CTACAAAAGA AGATAATGAA GACGATTTTA ATGAAAATAA AGACGTTTCC 2034

DvABCB1_FL_Sus AACATAGAGG CTACAAAAGA AGATAATGAA GACGATTTTA ATGAAAATAA AGACGTTTCC 2040

DvABCB1_3AR1 AACATAGAGG CTACAAAAGA AGATAATGAA GACGATTTTA ATGAAAATAA AGACGTTTCC 1314

DvABCB1_3AR2 ---------- ---------- ---------- ---------- ---------- ---------- 1003

DvABCB1_3AR3 AACATAGAGG CTACAAAAGA AGATAATGAA GACGATTTTA ATGAAAATAA AGACGTTTCC 2040

Consensus GTTTTTGAAA TATTGAAGAT GAATGCTCCA GAATGGCCTT ACATTTTATT TGCTGGTCTT 2094

DvABCB1_FL_Sus GTTTTTGAAA TATTAAAGAT GAATGCTCCA GAATGGCCTT ACATTTTATT TGCTGGTCTT 2100

DvABCB1_3AR1 GTTTTTGAAA TATTGAAGAT GAATGCTCCA GAATGGCCTT ACATTTTATT TGCTGGTCTT 1374

DvABCB1_3AR2 ---------- ---------- ---------- ---------- ---------- ---------- 1003

DvABCB1_3AR3 GTTTTTGAAA TATTGAAGAT GAATGCTCCA GAATGGCCTT ACATTTTATT TGCTGGTCTT 2100

Consensus GGATCAATTG TTGTTGGTTG TGGAATGCCC GTTTTTGCAG TCTTGTTTGG TAGCATTTTG 2154

DvABCB1_FL_Sus GGATCAATTG TTGTTGGTTG TGGAATGCCC GTTTTTGCAG TCTTGTTTGG TAGCATTTTG 2160

DvABCB1_3AR1 GGATCAATTG TTGTTGGTTG TGGAATGCCC GTTTTTGCAG TCTTGTTTGG TAGCATTTTG 1434

DvABCB1_3AR2 ---------- ---------- ---------- ---------- ---------- ---------- 1003

DvABCB1_3AR3 GGATCAATTG TTGTTGGTTG TGGAATGCCC GTTTTTGCAG TCTTGTTTGG TAGCATTTTG 2160

Consensus GGGACATTAG CAAATGGTGA TCCTGACTTT GTAAGATCTG AAACCAACAA ATATTGCTTA 2214

DvABCB1_FL_Sus GGGACATTAG CAAATGGTGA TCCTGACTTT GTAAGATCTG AAACCAACAA ATATTGCTTA 2220

DvABCB1_3AR1 GGGACATTAG CAAATGGTGA TCCTGACTTT GTAAGATCTG AAACCAACAA ATATTGCTTA 1494

DvABCB1_3AR2 ---------- ---------- ---------- ---------- ---------- ---------- 1003

DvABCB1_3AR3 GGGACATTAG CAAATGGTGA TCCTGACTTT GTAAGATCTG AAACCAACAA ATATTGCTTA 2220

Consensus TATTTTGTGC TGGGAGGACT CATAACTATG GTATCAGTAT TTACTCAGAT GTACCTGTTA 2274

DvABCB1_FL_Sus TATTTTGTGC TGGGAGGACT CATAACTATG GTATCAGTAT TTACTCAGAT GTACCTGTTA 2280

DvABCB1_3AR1 TATTTTGTGC TGGGAGGACT CATAACTATG GTATCAGTAT TTACTCAGAT GTACCTGTTA 1554

DvABCB1_3AR2 ---------- ---------- ---------- ---------- ---------- ---------- 1003

DvABCB1_3AR3 TATTTTGTGC TGGGAGGACT CATAACTATG GTATCAGTAT TTACTCAGAT GTACCTGTTA 2280

Consensus GGAATTGCTG GTGAAAAAAT GACAGAAAGA GTTAGGAGTC GATTATTCAA GGCAATGATA 2334

DvABCB1_FL_Sus GGAATTGCTG GTGAAAAAAT GACAGAAAGA GTTAGGAGTC GATTATTCAA GGCAATGATA 2340

DvABCB1_3AR1 GGAATTGCTG GTGAAAAAAT GACAGAAAGA GTTAGGAGTC GATTATTCAA GGCAATGATA 1614

DvABCB1_3AR2 ---------- ---------- ---------- ---------- ---------- ---------- 1003

DvABCB1_3AR3 GGAATTGCTG GTGAAAAAAT GACAGAAAGA GTTAGGAGTC GATTATTCAA GGCAATGATA 2340

Consensus TACCAGGAAA TAGGCTTCTT TGACAAGAAA ACAAATGGGG TTGGAGCGCT TTGTGCCAAA 2394

DvABCB1_FL_Sus TACCAGGAAA TAGGCTTCTT TGACAAGAAA ACAAATGGGG TTGGAGCGCT TTGTGCCAAA 2400

DvABCB1_3AR1 TACCAGGAAA TAGGCTTCTT TGACAAGAAA ACAAATGGGG TTGGAGCGCT TTGTGCCAAA 1674

DvABCB1_3AR2 ---------- ---------- ---------- ---------- ---------- ---------- 1003

DvABCB1_3AR3 TACCAGGAAA TAGGCTTCTT TGACAAGAAA ACAAATGGGG TTGGAGCGCT TTGTGCCAAA 2400

Consensus TTGTCTTCTG ATGCTTCTAA TATTCAAGGG GCAACGGGAA TTCGGGTTGG AACCATTCTT 2454

DvABCB1_FL_Sus TTGTCTTCTG ATGCTTCTAA TATTCAAGGG GCAACAGGAA TTCGGGTTGG AACCATTCTT 2460

DvABCB1_3AR1 TTGTCTTCTG ATGCTTCTAA TATTCAAGGG GCAACGGGAA TTCGGGTTGG AACCATTCTT 1734

DvABCB1_3AR2 ---------- ---------- ---------- ---------- ---------- ---------- 1003

DvABCB1_3AR3 TTGTCTTCTG ATGCTTCTAA TATTCAAGGG GCAACGGGAA TTCGGGTTGG AACCATTCTT 2460

Consensus CAATCAATAG CCACATTTTG CTTAGCAATT GGTCTTTCCA TGTATTATGA ATGGAAATTG 2514

DvABCB1_FL_Sus CAATCAATAG CCACATTTTG CTTAGCAATT GGTCTTTCCA TGTATTATGA ATGGAAATTG 2520

DvABCB1_3AR1 CAATCAATAG CCACATTTTG CTTAGCAATT GGTCTTTCCA TGTATTATGA ATGGAAATTG 1794

DvABCB1_3AR2 ---------- ---------- ---------- ---------- ---------- ---------- 1003

DvABCB1_3AR3 CAATCAATAG CCACATTTTG CTTAGCAATT GGTCTTTCCA TGTATTATGA ATGGAAATTG 2520

Consensus GGCTTAGTTA CAGCAGCTTT CACACCTGTT ATTTTGATCG CTATGTTCTT TGAAAGAAGA 2574

DvABCB1_FL_Sus GGTTTAGTTA CAGCAGCTTT CACACCTGTT ATTTTGATCG CTATGTTCTT TGAAAGAAGA 2580

DvABCB1_3AR1 GGCTTAGTTA CAGCAGCTTT CACACCTGTT ATTTTGATCG CTATGTTCTT TGAAAGAAGA 1854

DvABCB1_3AR2 ---------- ---------- ---------- ---------- ---------- ---------- 1003

DvABCB1_3AR3 GGCTTAGTTA CAGCAGCTTT CACACCTGTT ATTTTGATCG CTATGTTCTT TGAAAGAAGA 2580

Consensus AACACCAGAG GTGGAAACGA TTCAAGAGAT TCCGCACTAC AAAAATCAAC AAGGACTGCA 2634

DvABCB1_FL_Sus AACACCAGAG GTGGAAACGA TTCAAGAGAT TCCGCACTAC AAAAATCAAC AAGGACTGCA 2640

DvABCB1_3AR1 AACACCAGAG GTGGAAACGA TTCAAGAGAT TCCGCACTAC AAAAATCAAC AAGGACTGCA 1914

DvABCB1_3AR2 ---------- ---------- ---------- ---------- ---------- ---------- 1003

DvABCB1_3AR3 AACACCAGAG GTGGAAACGA TTCAAGAGAT TCCGCACTAC AAAAATCAAC AAGGACTGCA 2640

Consensus GTAGAAGCCG TTGGGAATAT ACGAACAGTT GCCTCCTTAG GCTTAGAAGA AAAGTTCCAG 2694

DvABCB1_FL_Sus GTAGAAGCCG TTGGGAATAT ACGAACGGTT GCCTCCTTGG GCTTAGAAGA AAAGTTCCAG 2700

DvABCB1_3AR1 GTAGAAGCCG TTGGGAATAT ACGAACAGTT GCCTCCTTAG GCTTAGAAGA AAAGTTCCAG 1974

DvABCB1_3AR2 ---------- ---------- ---------- ---------- ---------- ---------- 1003

DvABCB1_3AR3 GTAGAAGCCG TTGGGAATAT ACGAACAGTT GCCTCCTTAG GCTTAGAAGA AAAGTTCCAG 2700

Consensus CAGCTATATG AATCTGAGCT CATGCCACAT TATAAATCTT CGTTAAAAAC AGTTCACTGG 2754

DvABCB1_FL_Sus CAACTATATG AATCTGAGCT CATGCCACAT TATAAATCTT CGTTAAAAAC AGTTCACTGG 2760

DvABCB1_3AR1 CAGCTATATG AATCTGAGCT CATGCCACAT TATAAATCTT CGTTAAAAAC AGTTCACTGG 2034

DvABCB1_3AR2 ---------- ---------- ---------- ---------- ---------- ---------- 1003

DvABCB1_3AR3 CAGCTATATG AATCTGAGCT CATGCCACAT TATAAATCTT CGTTAAAAAC AGTTCACTGG 2760

Consensus AGAGCAATCG TGTTTGGTCT TTCCAGAAGT TTGTTGTTCT TTGCTTATGC TACCGCAATG 2814

DvABCB1_FL_Sus AGAGCAATCG TGTTTGGTCT TTCCAGAAGT TTGTTGTTCT TTGCTTATGC TACTGCAATG 2820

DvABCB1_3AR1 AGAGCAATCG TGTTTGGTCT TTCCAGAAGT TTGTTGTTCT TTGCTTATGC TACCGCAATG 2094

DvABCB1_3AR2 ---------- ---------- ---------- ---------- ---------- ---------- 1003

DvABCB1_3AR3 AGAGCAATCG TGTTTGGTCT TTCCAGAAGT TTGTTGTTCT TTGCTTATGC TACCGCAATG 2820

Consensus TACTACGGCG GATTTTTGAT AAGGGATGGA TTGCCATACG ATAGAGTGTT TAAGGTATCT 2874

DvABCB1_FL_Sus TATTACGGCG GATTTTTGAT AAGGGATGGA TTGCCATACG ATAGAGTATT TAAGGTATCT 2880

DvABCB1_3AR1 TACTACGGCG GATTTTTGAT AAGGGATGGA TTGCCATACG ATAGAGTGTT TAAGGTATCT 2154

DvABCB1_3AR2 ---------- ---------- ---------- ---------- ---------- ---------- 1003

DvABCB1_3AR3 TACTACGGCG GATTTTTGAT AAGGGATGGA TTGCCATACG ATAGAGTGTT TAAGGTATCT 2880

Consensus CAAGCACAAA TTATGGGGAC AGTTTCAATT GCCAACTCAC TGGCATTTTC TCCAAACTTT 2934

DvABCB1_FL_Sus CAAGCACAAA TTATGGGTAC AGTTTCAATT GCCAACTCAC TGGCATTTTC TCCAAACTTT 2940

DvABCB1_3AR1 CAAGCACAAA TTATGGGGAC AGTTTCAATT GCCAACTCAC TGGCATTTTC TCCAAACTTT 2214

DvABCB1_3AR2 ---------- ---------- ---------- ---------- ---------- ---------- 1003

DvABCB1_3AR3 CAAGCACAAA TTATGGGGAC AGTTTCAATT GCCAACTCAC TGGCATTTTC TCCAAACTTT 2940

Consensus GCTAAGGGTG TTGCAGCAGC AAAAAAAGTT AAAAGCTTCC TTTCAAGAAT TCCACTTATT 2994

DvABCB1_FL_Sus GCTAAGGGTG TTGCAGCAGC AAAAAAAGTT AAAAGCTTCC TTTCAAGAAT TCCACTTATT 3000

DvABCB1_3AR1 GCTAAGGGTG TTGCAGCAGC AAAAAAAGTT AAAAGCTTCC TTTCAAGAAT TCCACTTATT 2274

DvABCB1_3AR2 ---------- ---------- ---------- ---------- ---------- ---------- 1003

DvABCB1_3AR3 GCTAAGGGTG TTGCAGCAGC AAAAAAAGTT AAAAGCTTCC TTTCAAGAAT TCCACTTATT 3000

Consensus CGTGATTTAC CTTCTTCACG ACAAATGGTT AAGGCAAGTG GCAACTTCAG TTTTTCAGAA 3054

DvABCB1_FL_Sus CGTGATTTAC CTTCTTCACG ACAAATGGTT AAGGCAAGTG GCAACTTCAG TTTTTCAGAA 3060

DvABCB1_3AR1 CGTGATTTAC CTTCTTCACG ACAAATGGTT AAGGCAAGTG GCAACTTCAG TTTTTCAGAA 2334

DvABCB1_3AR2 ---------- ---------- ---------- ---------- ---------- ---------- 1003

DvABCB1_3AR3 CGTGATTTAC CTTCTTCACG ACAAATGGTT AAGGCAAGTG GCAACTTCAG TTTTTCAGAA 3060

Consensus ATCGAATTTA CATATCCAAC ACGACCCAAC GTACTAATTT TAAAAGGACT TAATTTGGAC 3114

DvABCB1_FL_Sus ATCGAATTTA CGTATCCAAC ACGACCCAAC GTACTAATTT TAAAAGGACT TAATTTGGAC 3120

DvABCB1_3AR1 ATCGAATTTA CATATCCAAC ACGACCCAAC GTACTAATTT TAAAAGGACT TAATTTGGAC 2394

DvABCB1_3AR2 ---------- ---------- ---------- ---------- ---------- ---------- 1003

DvABCB1_3AR3 ATCGAATTTA CATATCCAAC ACGACCCAAC GTACTAATTT TAAAAGGACT TAATTTGGAC 3120

Consensus ATTCTAAATG GAAAAACTGT AGCTCTAGTG GGAGAAAGTG GTTGCGGAAA ATCTACGATA 3174

DvABCB1_FL_Sus ATTCTAAATG GAAAAACTGT AGCTCTAGTG GGAGAAAGTG GTTGCGGAAA ATCTACGATA 3180

DvABCB1_3AR1 ATTCTAAATG GAAAAACTGT AGCTCTAGTG GGAGAAAGTG GTTGCGGAAA ATCTACGATA 2454

DvABCB1_3AR2 ---------- ---------- ---------- ---------- ---------- ---------- 1003

DvABCB1_3AR3 ATTCTAAATG GAAAAACTGT AGCTCTAGTG GGAGAAAGTG GTTGCGGAAA ATCTACGATA 3180

Consensus ATTCAACTCA TCGAGAGATT TTACGATCCT AGATCTGGTG AAGTAAAAAT GGATGGTGTA 3234

DvABCB1_FL_Sus ATTCAACTCA TCGAGAGATT TTACGATCCT AGATCTGGTG AAGTAAAAAT GGATGGTGTA 3240

DvABCB1_3AR1 ATTCAACTCA TCGAGAGATT TTACGATCCT AGATCTGGTG AAGTAAAAAT GGATGGTGTA 2514

DvABCB1_3AR2 ---------- ---------- ---------- ---------- ---------- ---------- 1003

DvABCB1_3AR3 ATTCAACTCA TCGAGAGATT TTACGATCCT AGATCTGGTG AAGTAAAAAT GGATGGTGTA 3240

Consensus GATTTAAAAG ATATATCGCT AGATTCGTTG AGGTCGCATA TGGGTATCGT ATCACAAGAA 3294

DvABCB1_FL_Sus GATTTAAAAG ATATATCACT AGATTCGTTG AGGTCGCATA TGGGTATAGT ATCACAAGAA 3300

DvABCB1_3AR1 GATTTAAAAG ATATATCGCT AGATTCGTTG AGGTCGCATA TGGGTATCGT ATCACAAGAA 2574

DvABCB1_3AR2 ---------- ---------- ---------- ---------- ---------- ---------- 1003

DvABCB1_3AR3 GATTTAAAAG ATATATCGCT AGATTCGTTG AGGTCGCATA TGGGTATCGT ATCACAAGAA 3300

Consensus CCAAATCTGT TCAATAAGTC TATTGCTGAA AATATTGCTT ATGGAGATAA TTCAAGGGAG 3354

DvABCB1_FL_Sus CCAAATCTGT TCAATAAGTC TATTGCTGAA AATATTGCTT ATGGAGATAA TTCAAGGGAG 3360

DvABCB1_3AR1 CCAAATCTGT TCAATAAGTC TATTGCTGAA AATATTGCTT ATGGAGATAA TTCAAGGGAG 2634

DvABCB1_3AR2 ---------- ---------- ---------- ---------- ---------- ---------- 1003

DvABCB1_3AR3 CCAAATCTGT TCAATAAGTC TATTGCTGAA AATATTGCTT ATGGAGATAA TTCAAGGGAG 3360

Consensus GTTTCTATGG ATGAGATAAT TAAAGCTGCT AAAAATGCCA ATATTCATAA TTTTATAACT 3414

DvABCB1_FL_Sus GTTTCTATGG ATGAGATAAT TAAAGCTGCT AAAAATGCCA ATATTCATAA TTTTATAACT 3420

DvABCB1_3AR1 GTTTCTATGG ATGAGATAAT TAAAGCTGCT AAAAATGCCA ATATTCATAA TTTTATAACT 2694

DvABCB1_3AR2 ---------- ---------- ---------- ---------- ---------- ---------- 1003

DvABCB1_3AR3 GTTTCTATGG ATGAGATAAT TAAAGCTGCT AAAAATGCCA ATATTCATAA TTTTATAACT 3420

Consensus GGATTACCGA AGGGATATGA AACAAAACTA GGAGAAAAGG CTGTTCAGTT GTCTGGTGGA 3474

DvABCB1_FL_Sus GGATTACCGA AGGGATATGA AACAAAACTA GGAGAAAAGG CTGTTCAGTT GTCTGGTGGA 3480

DvABCB1_3AR1 GGATTACCGA AGGGATATGA AACAAAACTA GGAGAAAAGG CTGTTCAGTT GTCTGGTGGA 2754

DvABCB1_3AR2 ---------- ---GATATGA AACAAAACTA GGAGAAAAGG CTGTTCAGTT GTCTGGTGGA 1050

DvABCB1_3AR3 GGATTACCGA AGGGATATGA AACAAAACTA GGAGAAAAGG CTGTTCAGTT GTCTGGTGGA 3480

Consensus CAAAAACAAA GAATTGCTAT TGCCAGGGCG TTAGTAAGAA ATCCAAAAGT TTTACTCCTT 3534

DvABCB1_FL_Sus CAAAAACAAA GAATTGCTAT TGCCAGAGCG TTAGTAAGAA ATCCAAAAGT TTTACTCCTT 3540

DvABCB1_3AR1 CAAAAACAAA GAATTGCTAT TGCCAGGGCG TTAGTAAGAA ATCCAAAAGT TTTACTCCTT 2814

DvABCB1_3AR2 CAAAAACAAA GAATTGCTAT TGCCAGGGCG TTAGTAAGAA ATCCAAAAGT TTTACTCCTT 1110

DvABCB1_3AR3 CAAAAACAAA GAATTGCTAT TGCCAGGGCG TTAGTAAGAA ATCCAAAAGT TTTACTCCTT 3540

Consensus GATGAAGCCA CATCTGCACT GGACACTGAA AGTGAGAAGG TTGTTCAAGA AGCTCTAGAT 3594

DvABCB1_FL_Sus GATGAAGCCA CATCTGCACT AGACACTGAA AGTGAGAAGG TTGTTCAAGA AGCTCTAGAT 3600

DvABCB1_3AR1 GATGAAGCCA CATCTGCACT GGACACTGAA AGTGAGAAGG TTGTTCAAGA AGCTCTAGAT 2874

DvABCB1_3AR2 GATGAAGCCA CATCTGCACT GGACACTGAA AGTGAGAAGG TTGTTCAAGA AGCTCTAGAT 1170

DvABCB1_3AR3 GATGAAGCCA CATCTGCACT GGACACTGAA AGTGAGAAGG TTGTTCAAGA AGCTCTAGAT 3600

Consensus CAAGCTAAAA AAGGAAGGAC ATGTGTTACG ATTGCCCACA GATTAACAAC CATACAAGAT 3654

DvABCB1_FL_Sus CAAGCTAAAA AAGGAAGGAC ATGTGTTACG ATTGCCCACA GATTAACAAC CATACAAGAT 3660

DvABCB1_3AR1 CAAGCTAAAA AAGGAAGGAC ATGTGTTACG ATTGCCCACA GATTAACAAC CATACAAGAT 2934

DvABCB1_3AR2 CAAGCTAAAA AAGGAAGGAC ATGTGTTACG ATTGCCCACA GATTAACAAC CATACAAGAT 1230

DvABCB1_3AR3 CAAGCTAAAA AAGGAAGGAC ATGTGTTACG ATTGCCCACA GATTAACAAC CATACAAGAT 3660

Consensus GCAGACTTGA TATGCGTAGT AGCAAATGGT GTAATTGCTG AATCTGGAAG CCATCAAGAA 3714

DvABCB1_FL_Sus GCAGACTTGA TATGCGTAGT AGCAAATGGT GTAATTGCTG AATCTGGAAG CCATCAAGAA 3720

DvABCB1_3AR1 GCAGACTTGA TATGCGTAGT AGCAAATGGT GTAATTGCTG AATCTGGAAG CCATCAAGAA 2994

DvABCB1_3AR2 GCAGACTTGA TATGCGTAGT AGCAAATGGT GTAATTGCTG AATCTGGAAG CCATCAAGAA 1290

DvABCB1_3AR3 GCAGACTTGA TATGCGTAGT AGCAAATGGT GTAATTGCTG AATCTGGAAG CCATCAAGAA 3720

Consensus CTTCTGCAAA AGGAGGGACT GTACTACAAA TTATATACTC AAAAAACGTA A 3765

DvABCB1_FL_Sus CTTCTGCAAA AGGAGGGACT GTACTACAAA TTATATACTC AAAAAACGTA A 3771

DvABCB1_3AR1 CTTCTGCAAA AGGAGGGACT GTACTACAAA TTATATACTC AAAAAACGTA A 3045

DvABCB1_3AR2 CTTCTGCAAA AGGAGGGACT GTACTACAAA TTATATACTC AAAAAACGTA A 1341

DvABCB1_3AR3 CTTCTGCAAA AGGAGGGACT GTACTACAAA TTATATACTC AAAAAACGTA A 3771

**Supplementary Figure 3. Nucleotide sequence alignment of *DvABCB1* sequences from susceptible and mCry3A-selected WCR.** *DvABCB1* susceptible sequence (DvABCB1-FL-Sus) aligned with DvABCB1_3AR1, DvABCB1_3AR2, and DvABCB1_3AR3 (full length) RT-PCR amplicons sequences from mCry3A-selected WCR. Alignment was generated with Geneious Alignment using Global alignment with free end gaps in Geneious Prime.

**
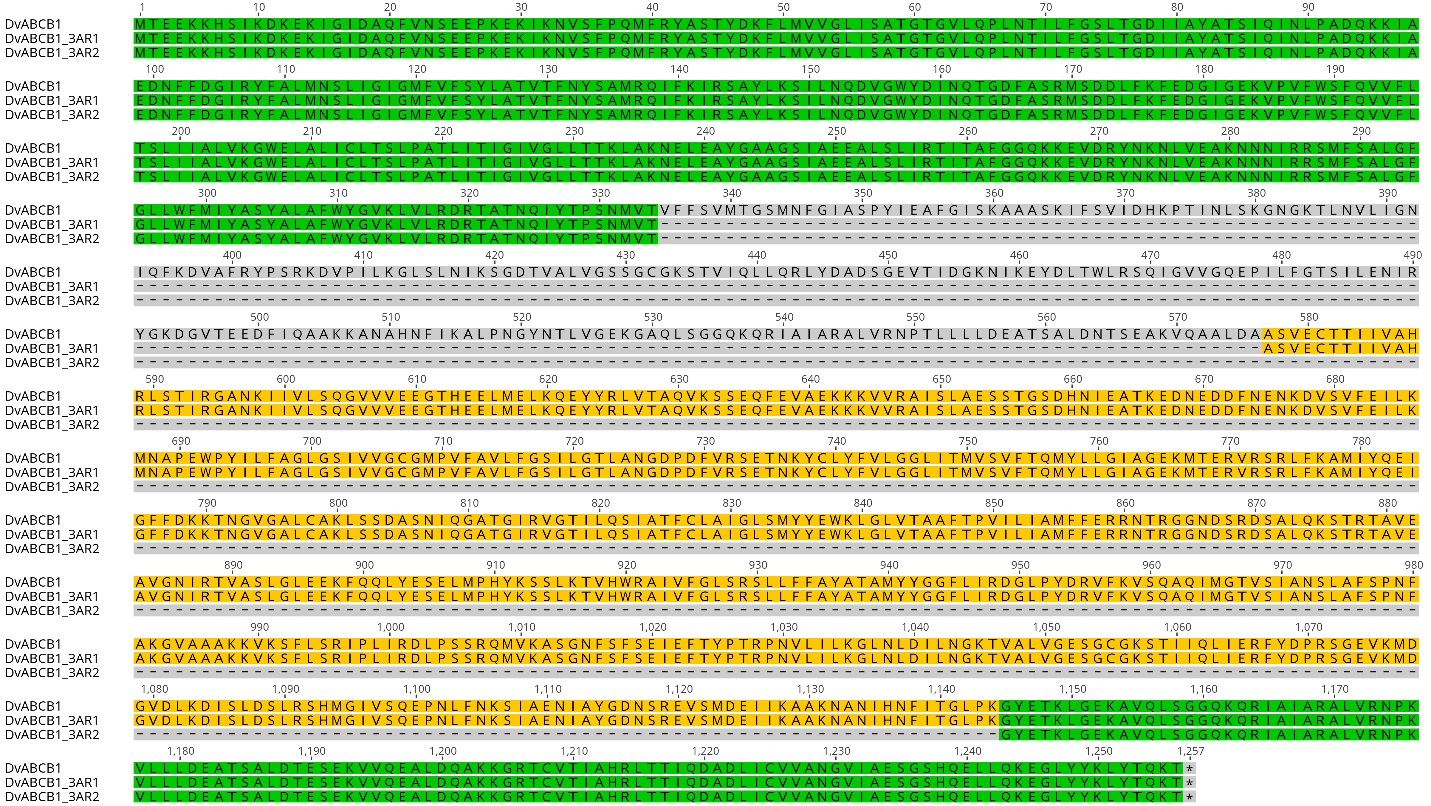
**

**Supplementary Figure 4. Protein alignment of DvABCB1 sequences from susceptible and mCry3A-selected WCR.** DvABCB1 sequence aligned with DvABCB1_3AR1 and DvABCB1_3AR2 translated RT-PCR amplicons sequences from mCry3A-selected WCR. Alignment was generated with Geneious Alignment using Blosum62 score matrix in Geneious Prime. Deletion locations in mCry3A-selected WCR DvABCB1 sequences: amino acids 335-576 in 3AR1 and amino acids 335-1144 in 3AR2. Highlighted amino acids identities: Green: 100%; Yellow: 60-80%; Gray: <60%. Note that the DvABCB1 sequence obtained from the amplicon sequences from the susceptible WCR strain differs from DvABCB1 aligned here at amino acid position 501 (F to V) due to a non-silent SNP.

**Supplementary Figure 5. *DvABCB1* transcript levels in WCR susceptible and resistant strains.** The transcript levels of *DvABCB1* were quantified in pulled guts of 3rd instar WCR larvae, 10 guts per sample, 3 samples per strain. qRT-PCR assay was done as described in Materials and Methods with 2 replicates per sample. Relative expression of *DvABCB1* by qRT-PCR assay is shown for susceptible and resistant strains using *DvRPS10* as a reference and normalized to one replicate in susceptible sample (mean ± SE; n = 6 per strain). *DvABCB1* expression (F _(1, 10)_ = 70.30, *p*-value < 0.0001).
